# Inhibition of BAP1 Sensitizes Gastric Cancer Cells to 5-Fluorouracil by Promoting BIRC5 Degradation

**DOI:** 10.3390/ph19101593

**Published:** 2026-10-08

**Authors:** In-Ho Jeong, Yu-Jin Park, Min-Jee Kim, Eun Ju Lee, Byung Sik Kim, Hee-Sung Kim, Peter Chang-Whan Lee

**Affiliations:** 1 Department of Biochemistry and Molecular Biology, Asan Medical Center, University of Ulsan College of Medicine, Seoul 05505, Republic of Korea; d4inno@naver.com (I.-H.J.); yj041801@gmail.com (Y.-J.P.); pradoxwh@naver.com (M.-J.K.); eunju8527@gmail.com (E.J.L.); 2 Department of General Surgery, Hallym University College of Medicine, Hallym Medical Center, Anyang 14068, Republic of Korea; amcgs009@gmail.com

**Keywords:** BAP1, BIRC5, 5-FU, gastric cancer, apoptosis

## Abstract

**Background/Objectives:** Gastric cancer is an aggressive malignancy with poor prognosis, and acquired resistance to 5-fluorouracil (5-FU) remains a major therapeutic challenge. BRCA1-associated protein-1 (BAP1), a deubiquitinating enzyme, has been implicated in tumorigenesis; however, its role in gastric cancer and 5-FU resistance remains unclear. This study investigated the functional and molecular roles of BAP1 in gastric cancer progression and chemoresistance. **Methods:** BAP1 expression and its association with patient prognosis were evaluated in gastric cancer tissues. The effects of BAP1 depletion on cancer cell proliferation, migration, invasion, and apoptosis were examined. The interaction between BAP1 and BIRC5 (Survivin) and the effects of BAP1-mediated deubiquitination on BIRC5 stability were investigated. The therapeutic effect of the BAP1 inhibitor iBAP-II was further assessed in 5-FU resistant gastric cancer cells. **Results:** BAP1 was significantly upregulated in gastric cancer tissues and associated with poor prognosis. BAP1 knockdown suppressed proliferation, migration, and invasion. Further analysis revealed that BAP1 interacted with and stabilized BIRC5 through deubiquitination, thereby suppressing apoptosis. BAP1 and BIRC5 were markedly upregulated in 5-FU resistant cells, whereas iBAP-II treatment enhanced 5-FU sensitivity and increased caspase-3 and PARP cleavage. **Conclusions:** BAP1 promotes gastric cancer progression and 5-FU resistance by stabilizing BIRC5. Targeting the BAP1–BIRC5 axis may represent a promising strategy for overcoming 5-FU resistance and improving therapeutic efficacy in gastric cancer.

## 1. Introduction

Gastric cancer remains a major global health burden and continues to be one of the leading causes of cancer-related deaths worldwide. According to the most recent global cancer statistics reported by the International Agency for Research on Cancer through the GLOBOCAN database, an estimated 1,089,103 new gastric cancer cases and 768,793 deaths occurred globally in 2020, making gastric cancer the fifth most commonly diagnosed cancer and the fourth leading cause of cancer-related death worldwide [1]. The disease burden is particularly high in East Asian countries, including Korea, Japan, and China, where incidence rates remain among the highest globally. Despite improvements in surgical techniques and systemic treatment strategies, the prognosis of patients with advanced gastric cancer remains poor due to frequent metastasis and resistance to chemotherapy [2].

Fluoropyrimidine-based chemotherapy remains the backbone of systemic treatment for gastric cancer [3]. Among these agents, 5-Fluorouracil (5-FU) is widely used either as a single agent or in combination regimens such as FOLFOX, CAPOX, or FP therapy [4,5,6]. The anticancer activity of 5-FU primarily involves inhibition of thymidylate synthase and disruption of DNA synthesis, leading to impaired tumor cell proliferation and apoptosis [7]. However, the clinical benefit of 5-FU is frequently limited by the development of acquired chemoresistance during treatment. The emergence of 5-FU resistant tumor cells significantly reduces treatment efficacy and contributes to disease progression and poor patient outcomes [8]. Therefore, identifying the molecular mechanisms that regulate 5-FU resistance is critical for developing new therapeutic strategies to improve the efficacy of chemotherapy in gastric cancer.

The Ubiquitin–Proteasome System (UPS) plays a central role in maintaining intracellular protein homeostasis by regulating the ubiquitination and degradation of cellular proteins. In this system, proteins tagged with ubiquitin chains are recognized and degraded by the proteasome, whereas deubiquitinases (DUBs) remove ubiquitin moieties from target proteins and thereby stabilize their expression [9]. Increasing evidence suggests that dysregulation of the UPS contributes to tumor progression, metastasis, and resistance to anticancer therapies [10]. Because DUBs control the stability of numerous oncogenic and tumor-suppressive proteins, they have recently emerged as promising therapeutic targets in cancer [11,12]. Among DUBs, BRCA1-associated protein-1 (BAP1) is a ubiquitin C-terminal hydrolase belonging to the deubiquitinase family and participates in multiple cellular processes, including chromatin remodeling, DNA damage repair, and transcriptional regulation [13]. Aberrant expression or mutation of BAP1 has been reported in various cancers, suggesting its important role in tumor biology [14]. Although BAP1 has been predominantly characterized as a tumor suppressor gene in mesothelioma and uveal melanoma, where loss-of-function mutations are frequently observed, emerging evidence suggests that BAP1 may exert context-dependent, oncogenic functions in specific epithelial malignancies. The molecular basis for this dual role likely reflects cancer type-specific transcriptional programs, co-mutational landscapes, and substrate availability.

Previous work reported a link between BAP1 and anti-apoptotic signaling in melanoma [15], and bioinformatic analysis of TCGA gastric cancer datasets revealed a positive correlation between BAP1 and BIRC5 expression levels. Given that BIRC5 is a major IAP family member frequently overexpressed in gastrointestinal cancers and strongly associated with chemotherapy resistance, we hypothesized that BAP1 may directly regulate BIRC5 protein stability through its deubiquitinase activity. BIRC5 is frequently overexpressed in a wide range of malignancies and is associated with tumor aggressiveness, poor prognosis, and resistance to anticancer therapies [16,17]. By inhibiting caspase activation and promoting mitotic progression, BIRC5 enhances tumor cell survival under stress conditions, including exposure to chemotherapeutic agents. Stabilization of BIRC5 protein therefore represents an important mechanism by which cancer cells evade apoptosis and develop resistance to chemotherapy [18,19].

In the present study, we investigated the role of BAP1 in gastric cancer progression and chemoresistance. We found that BAP1 expression is significantly elevated in gastric cancer tissues and promotes tumorigenic properties in gastric cancer cells. Moreover, BAP1 interacts with and stabilizes BIRC5 through deubiquitination, thereby suppressing apoptosis. Importantly, pharmacological inhibition of BAP1 using the selective inhibitor iBAP-II markedly enhances the sensitivity of gastric cancer cells to 5-FU, particularly in 5-FU resistant cells. These findings suggest that targeting BAP1 may represent a promising strategy that sensitizes 5-FU resistant gastric cancer cells to 5-FU and improve the therapeutic efficacy of 5-FU-based chemotherapy in gastric cancer.

## 2. Results

### 2.1. BAP1 Is Overexpressed in Gastric Cancer Tissues and Correlates with Poor Prognosis

**Figure 1:**
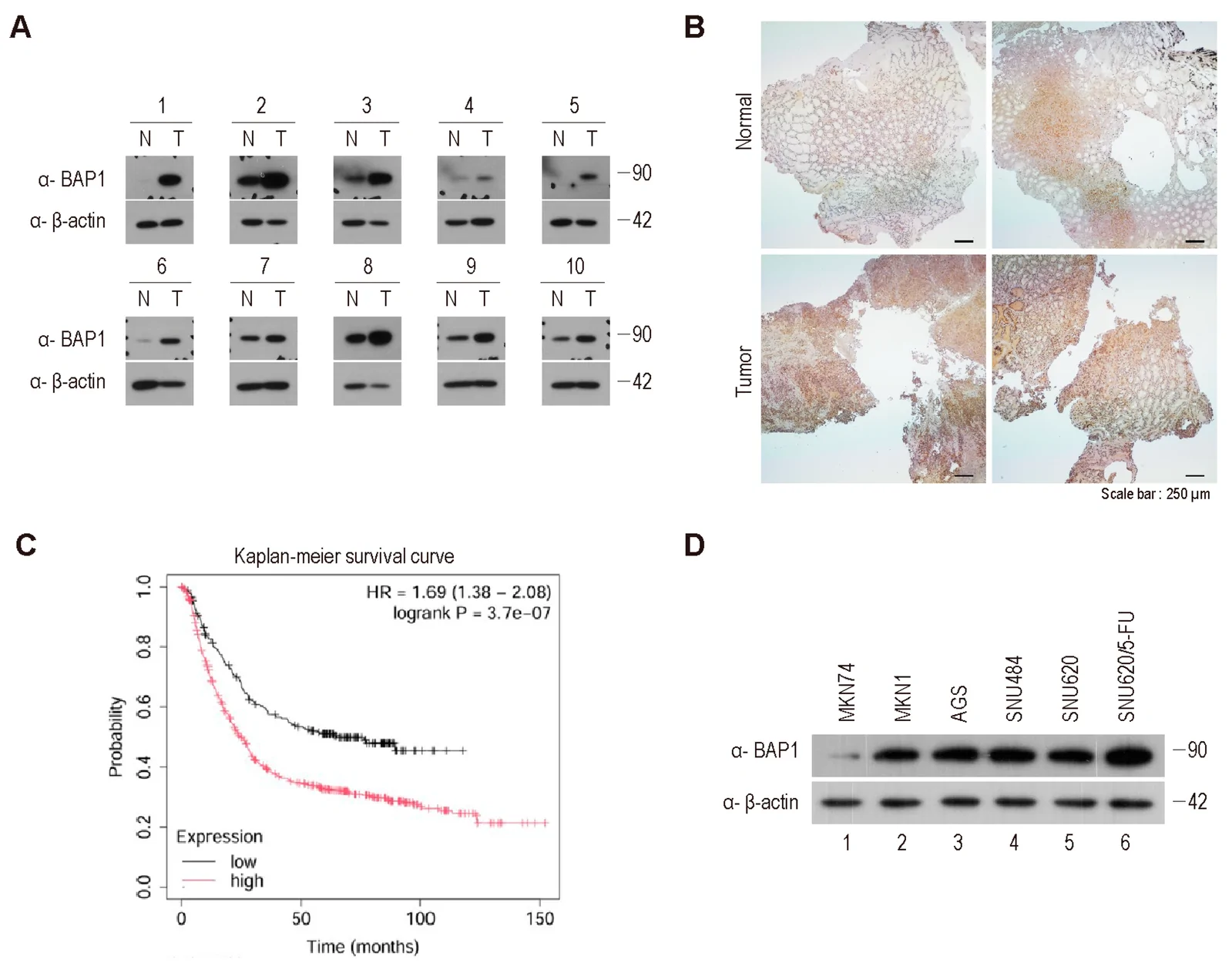
**BAP1 protein is overexpressed in the tissues of gastric cancer patients and is associated with a poor prognosis.** (**A**) Representative Western blot results of 20 paired adjacent normal gastric tissues (N) and their gastric cancer tissues (T). Each tissue was lysed by RIPA buffer and blotted with the BAP1 and β-actin antibodies. Representative Western blots from 10 of the 20 independent patient pairs are shown. (**B**) Representative IHC images on gastric cancer tissues with BAP1 antibody were obtained by a light microscope at 40× magnification. (**C**) Kaplan–Meier analysis of overall survival according to BAP1 expression (probe 201419_at) in a pooled gastric cancer microarray cohort (Kaplan–Meier plotter, *n* = 875; cut-point selected by the tool’s optimisation function). HR 1.69 (95% CI 1.38–2.08), log-rank *p* < 0.001. (**D**) Western blot images of BAP1 protein levels from several gastric cancer cell lines and β-actin was used for an internal control.

To investigate the potential involvement of BAP1 in gastric cancer, we first examined BAP1 protein expression in clinical gastric cancer specimens. Western blot analysis of 20 paired samples revealed that BAP1 protein levels were markedly elevated in tumor tissues compared with adjacent normal gastric tissues (Figure 1A and Figure S1A). Immunohistochemical staining further confirmed strong BAP1 expression in gastric cancer tissues (Figure 1B and Figure S1B).

To evaluate the clinical relevance of BAP1 expression, we analysed overall survival in a pooled gastric cancer microarray cohort using the Kaplan–Meier plotter (*n* = 875; probe 201419_at). High BAP1 expression was associated with significantly shorter overall survival (HR 1.69, 95% CI 1.38–2.08, log-rank *p* = 3.7 × 10^−7^; Figure 1C), and the association was preserved with a pre-specified median split (HR 1.49, 95% CI 1.25–1.76, *p* = 4.8 × 10^−6^). Because multivariable Cox regression is not available in this resource, we repeated the analysis within strata of stage, nodal status, sex and Lauren classification; the association was consistent in direction and magnitude across strata, although it was not evident in diffuse-type tumors (HR 1.08, 95% CI 0.77–1.51, *p* = 0.671) (Supplementary Figure S8 and Table S2B).

### 2.2. Knockdown of BAP1 Suppresses Tumorigenic Properties of Gastric Cancer Cells

**Figure 2:**
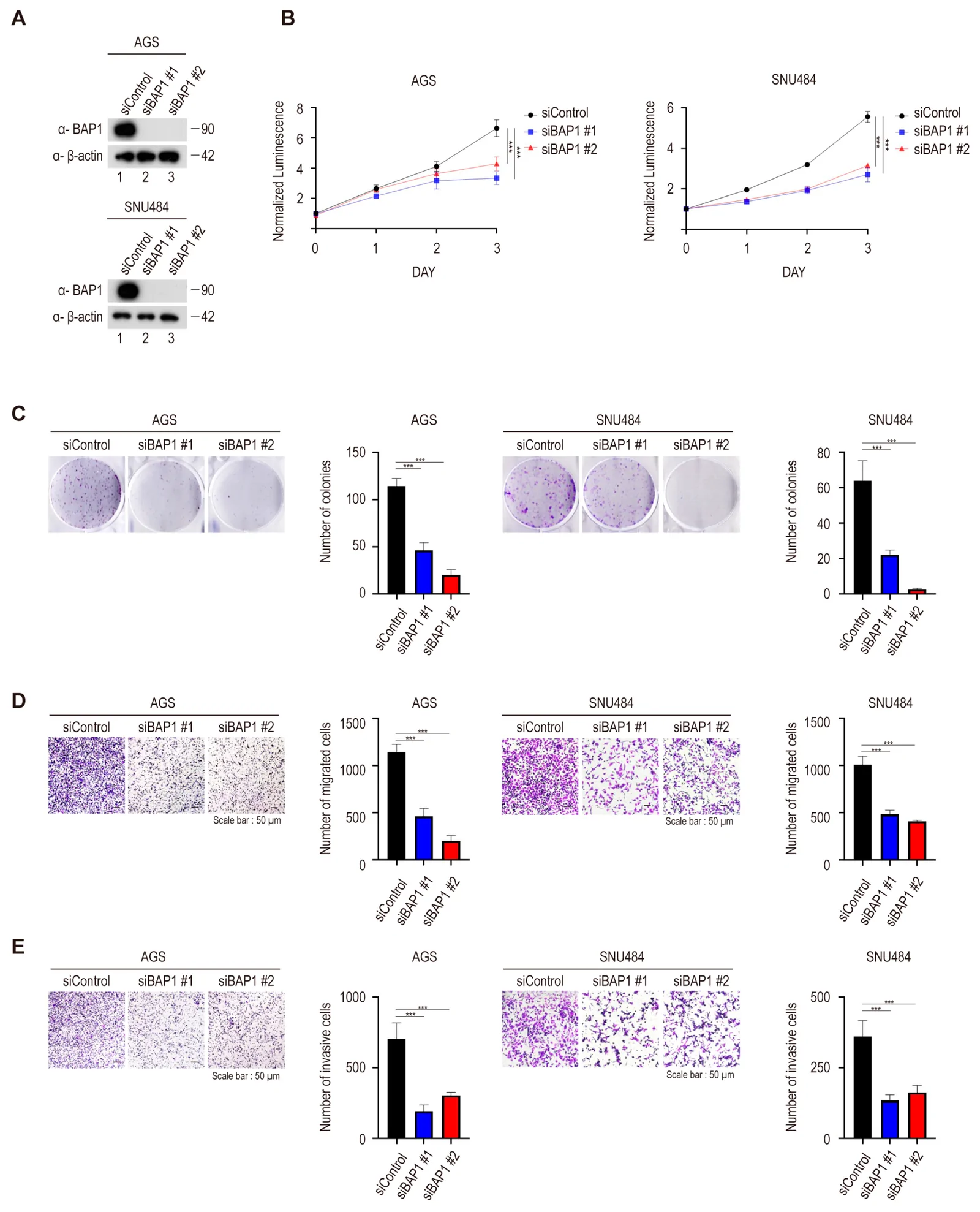
BAP1 protein knockdown inhibits tumorigenesis in gastric cancer cells. (**A**) Western blot analysis of BAP1 following siRNA transfection in AGS and SNU484 cells. (**B**) Effects of BAP1 knockdown on the viability of AGS and SNU484 cells. Cell viability was monitored for 3 days using a CellTiter-Glo luminescent assay (normalized to control siRNA at day 0). Data are mean ± SD of *n* = 5 independent experiments. Statistical significance was determined by two-way repeated-measures ANOVA (treatment × time interaction: AGS, F(6,24) = 83.4, *p* < 0.001; SNU484, F(6,24) = 62.3, *p* < 0.001), followed by one-way ANOVA with Dunnett’s post hoc test at each time point versus control siRNA. *** *p* < 0.001. (**C**) Effects of BAP1 knockdown on the colony formation of AGS and SNU484 cells. Cells transfected with control siRNA and BAP1 siRNAs were cultured for 10 days and stained with crystal violet to visualize. Data are mean ± SD of *n* = 3 independent experiments; one-way ANOVA with Dunnett’s post hoc test (AGS, F(2,6) = 221.8; SNU484, F(2,6) = 126.7; both *p* < 0.001). *** *p* < 0.001 versus control siRNA. (**D**) Effects of BAP1 knockdown on the migration of AGS and SNU484 using transwell inserts. Migration capacity was validated after a 24-h incubation. Data are mean ± SD of *n* = 3 independent experiments; one-way ANOVA with Dunnett’s post hoc test (AGS, F(2,6) = 73.3; SNU484, F(2,6) = 182.0; both *p* < 0.001). *** *p* < 0.001 versus control siRNA. (**E**) Effects of the BAP1 knockdown on cell invasion. AGS and SNU484 cells using transwell inserts containing Matrigel-coated membranes. Invasive capacity was validated after a 24-h incubation. Data are mean ± SD of *n* = 3 independent experiments; one-way ANOVA with Dunnett’s post hoc test (AGS, F(2,6) = 85.3; SNU484, F(2,6) = 91.5; both *p* < 0.001). *** *p* < 0.001 versus control siRNA.

To determine the functional role of BAP1 in gastric cancer cells, we performed loss-of-function experiments using siRNA-mediated knockdown in AGS and SNU484 cells (Figure 2A). Cell viability assays demonstrated that depletion of BAP1 significantly reduced cell proliferation compared with control cells (Figure 2B and Figure S2). Two-way ANOVA revealed a significant treatment × time interaction in both cell lines (both *p* < 0.001), and post hoc comparisons confirmed significant reductions at each time point (Supplementary Table S1). Consistent with that, colony formation assays revealed a marked decrease in clonogenic survival following BAP1 knockdown (Figure 2C). In addition, transwell migration and invasion assays showed that suppression of BAP1 significantly impaired both migratory and invasive capacities of gastric cancer cells (Figure 2D,E). These results indicate that BAP1 plays an important role in promoting tumorigenic characteristics of gastric cancer cells.

### 2.3. BAP1 Regulates BIRC5 Stability Through the Ubiquitin–Proteasome System

**Figure 3:**
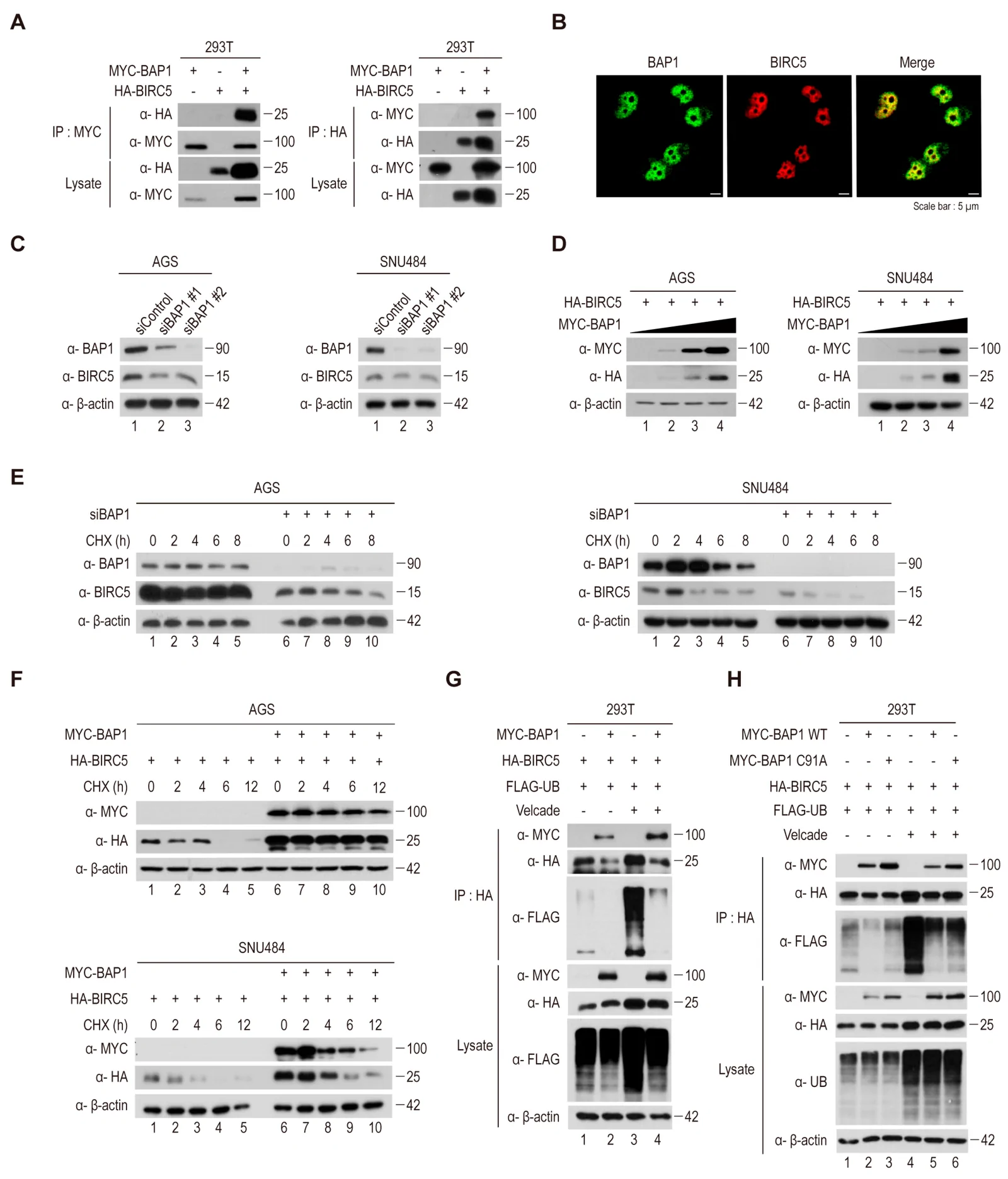
BAP1 regulates BIRC5 through the ubiquitin proteasome system. (**A**) Interaction between BAP1 and BIRC5 analyzed by immunoprecipitation and immunoblotting in HEK293T cells. HEK293T cells transfected with MYC-BAP1 and HA-BIRC5 plasmids were lysed and immunoprecipitated using MYC- or HA- antibodies-conjugated beads. (**B**) Immunofluorescence assay of BAP1 and BIRC5 protein expression in AGS cells using goat anti-mouse secondary antibody with Alexa Fluor™ 488 and goat anti-rabbit secondary antibody with Alexa Fluor™ 594. (**C**) BIRC5 protein levels in AGS and SNU484 cells following BAP1 knockdown. After 72 h incubation, cells were lysed and immunoblotted with the indicated antibodies. (**D**) HA-BIRC5 accumulation by MYC-BAP1 overexpression in AGS and SNU484 cells. Cells were transfected with a fixed amount (1 µg) of HA-BIRC5 and a gradient (from 1 to 3 µg) of MYC-BAP1 constructs. After 48 h incubation, cells were lysed and immunoblotted with the indicated antibodies. (**E**,**F**) BIRC5 protein stability assessed by cycloheximide chase assay with BAP1 knockdown (**E**) or overexpression (**F**). (**G**,**H**) Ubiquitination of BIRC5 analyzed by immunoprecipitation. HEK 293T cells were transfected with mixtures of plasmids expressing HA-BIRC5, MYC-BAP1 WT or C91A mutant and FLAG-Ubiquitin for 48 h. The cells were treated with Velcade (50 ng/ml) for 6 h prior to harvesting. Cell lysates were immunoprecipitated with HA beads, and immune complexes were immunoblotted with the indicated antibodies. All immunoprecipitation, immunofluorescence, cycloheximide chase and ubiquitination experiments are representative of *n* = 3 independent experiments.

To identify the downstream effectors responsible for the pro-tumorigenic functions of BAP1, we next sought to determine whether BAP1 regulates key apoptosis suppressors. We focused on BIRC5, a well-established IAP family member frequently co-overexpressed with oncogenic DUBs in gastrointestinal cancers. Co-immunoprecipitation assays demonstrated that BAP1 physically interacts with BIRC5 in HEK293T cells (Figure 3A). Immunofluorescence analysis further revealed colocalization of BAP1 and BIRC5 in gastric cancer cells (Figure 3B). Silencing of BAP1 markedly reduced BIRC5 protein levels in AGS and SNU484 cells (Figure 3C), whereas overexpression of BAP1 increased BIRC5 accumulation in a dose-dependent manner (Figure 3D). Cycloheximide chase assays showed that BAP1 knockdown significantly shortened the half-life of BIRC5 protein, while BAP1 overexpression substantially prolonged BIRC5 stability (Figure 3E,F and Figure S3). To further determine whether the reduced BIRC5 stability following BAP1 depletion is mediated by proteasomal degradation, cells were treated with the proteasome inhibitor, Velcade. Velcade treatment restored BIRC5 protein levels reduced by BAP1 knockdown, supporting that BAP1 maintains BIRC5 stability by protecting it from proteasomal degradation (Supplementary Figure S4).

Furthermore, ubiquitination assays demonstrated that wild-type BAP1 decreased the ubiquitination level of BIRC5, whereas the catalytically inactive BAP1 mutant (C91A) failed to do so (Figure 3G,H). These findings indicate that BAP1 stabilizes BIRC5 by removing ubiquitin chains and preventing its proteasomal degradation.

### 2.4. BAP1 Inhibition Induces Apoptosis Through Downregulation of BIRC5

**Figure 4:**
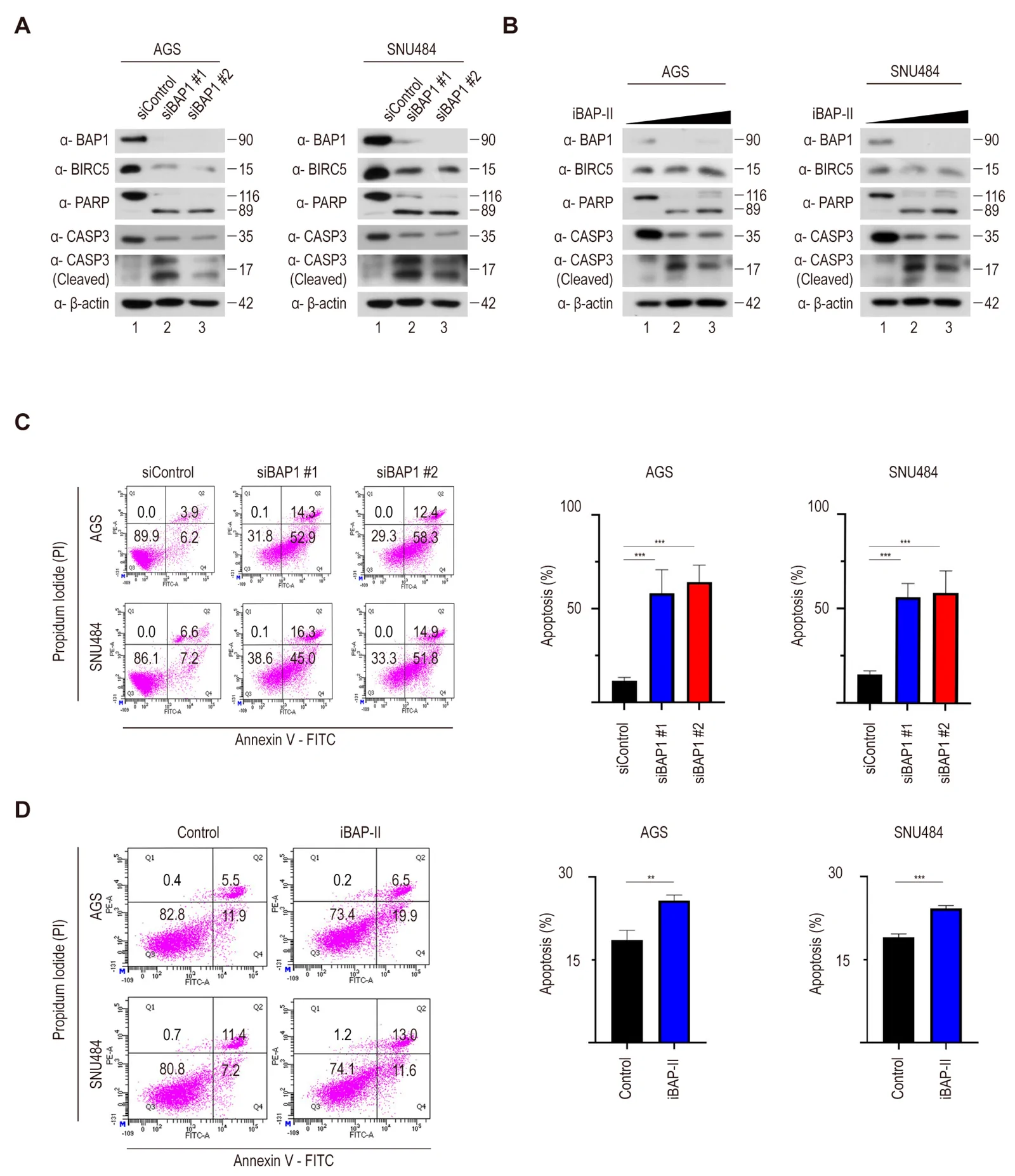
BAP1 knockdown induces apoptosis by reducing BIRC5 expression. (**A**) Western blot analysis of apoptotic markers following BAP1 knockdown in AGS and SNU484 cells. After 72 h incubation, the cells were lysed and immunoblotted with the indicated antibodies. Western blots are representative of *n* = 3 independent experiments. (**B**) Western blot analysis of apoptotic markers following iBAP-II treatment (0 µM, 48 µM, 64 µM) in AGS and SNU484 cells. After 24 h incubation, the cells were lysed and immunoblotted with the indicated antibodies. Western blots are representative of *n* = 3 independent experiments. (**C**) BAP1 knockdown-induced apoptosis was confirmed by Flow cytometry analysis. AGS and SNU484 cells were transfected with siBAP1 for 72 h, and then stained using Annexin V FITC Apoptosis detection kit. Data are mean ± SD of *n* = 3 independent experiments; one-way ANOVA with Dunnett’s post hoc test (AGS, F(2,6) = 49.0, *p* < 0.001; SNU484, F(2,6) = 55.4, *p* < 0.001). *** *p* < 0.001 versus control siRNA. (**D**) iBAP-II treatment-induced apoptosis was confirmed by Flow cytometry analysis. AGS and SNU484 cells were treated with iBAP-II (48 µM) for 24 h, and then stained using Annexin V FITC Apoptosis detection kit. Data are mean ± SD of *n* = 3 independent experiments. As only two groups were compared, significance was assessed by a two-sided Student’s *t*-test (AGS, *p* = 0.002; SNU484, *p* < 0.001). ** *p* < 0.01, *** *p* < 0.001.

Because BIRC5 functions as a key inhibitor of apoptosis, we next examined whether BAP1 influences apoptotic signaling. Knockdown of BAP1 significantly increased the expression of cleaved caspase-3 and cleaved PARP in gastric cancer cells (Figure 4A and Figure S5), suggesting activation of apoptosis pathways. Similarly, treatment with the selective BAP1 inhibitor iBAP-II produced comparable effects, leading to increased levels of apoptotic markers (Figure 4B). Flow cytometry analysis further confirmed that both BAP1 knockdown and pharmacological inhibition significantly increased the proportion of apoptotic cells (Figure 4C,D). These results demonstrate that inhibition of BAP1 promotes apoptosis in gastric cancer cells through destabilization of BIRC5.

### 2.5. Targeting the BAP1–BIRC5 Axis Sensitizes Gastric Cancer Cells to 5-FU

**Figure 5:**
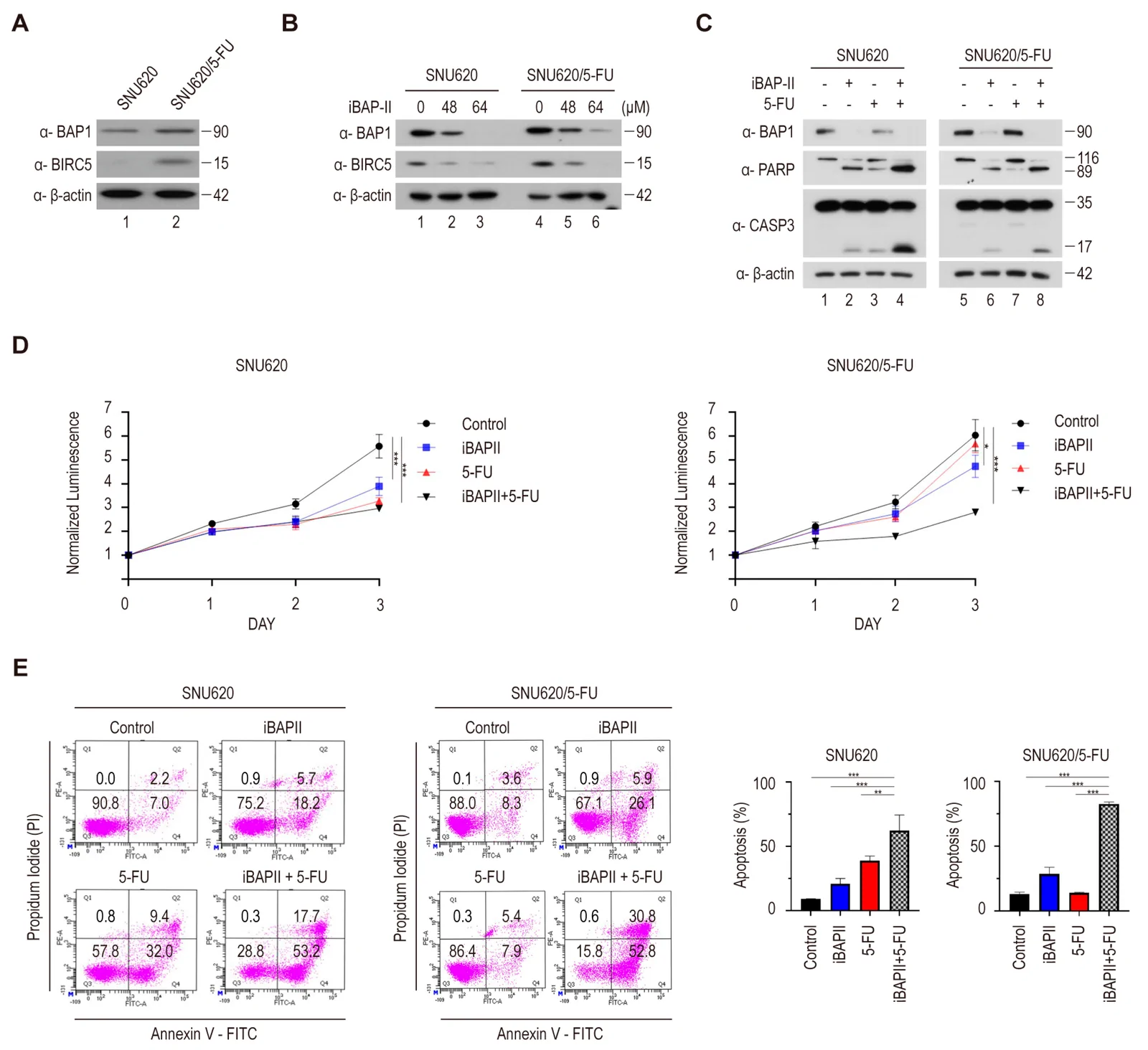
The BAP1-BIRC5 axis affects 5-FU resistance in gastric cancer. (**A**) Protein levels of BAP1 and BIRC5 in SNU620 and SNU620/5-FU cells. (**B**) Effects of iBAP-II on BAP1 and BIRC5 protein levels in SNU620 and SNU620/5-FU cells. Cells were treated with iBAP-II (0 µM, 48 µM, 64 µM) for 24 h and then harvested for following Western blot analysis. (**C**) SNU620 and SNU620/5-FU cells were treated with iBAP-II (48 µM) and 5-FU (3 µM) for 24 h prior to harvesting. Cell lysates were immunoblotted with the indicated antibodies. (**D**) Effect of combined iBAP-II and 5-FU treatment on the viability of SNU620 and SNU620/5-FU cells. 10 µM of iBAP-II and 3 µM of 5-FU was treated and cell viability was monitored for 3 days using a CellTiter-Glo luminescent assay (normalized to control wild-type cells at day 0). Data are mean ± SD of *n* = 3–5 independent experiments (exact *n* per group in Supplementary Table S1). Two-way ANOVA showed a significant treatment × time interaction in both lines (SNU620, F(9,52) = 26.9; SNU620/5-FU, F(9,56) = 28.8; both *p* < 0.001), followed by one-way ANOVA with Tukey’s HSD post hoc test at each time point. * *p* < 0.05, ** *p* < 0.01, *** *p* < 0.001. (**E**) iBAP-II and 5-FU treatment-induced apoptosis was confirmed by Flow cytometry analysis. SNU620 and SNU620/5-FU cells were treated with iBAP-II (48 µM) and 5-FU (3 µM) for 24 h prior to harvesting, and then stained using Annexin V FITC Apoptosis detection kit. Data are mean ± SD of *n* = 3 independent experiments; one-way ANOVA with Tukey’s HSD post hoc test (SNU620, F(3,8) = 70.4, *p* < 0.001; SNU620/5-FU, F(3,8) = 795.3, *p* < 0.001). * *p* < 0.05, ** *p* < 0.01, *** *p* < 0.001.

To determine whether BAP1 contributes to chemoresistance, we compared BAP1 and BIRC5 expression between parental SNU620 cells and their 5-FU resistant counterpart (SNU620/5-FU). Before the experiments, we compared the 5-FU IC_50_; values of SNU620 and SNU620/5-FU cells and found that the IC_50_; of SNU620/5-FU cells was approximately seven-fold higher than that of parental SNU620 cells (Supplementary Figure S10). As a result, both proteins were markedly elevated in the resistant cells (Figure 5A). Treatment with the BAP1 inhibitor iBAP-II significantly reduced BAP1 and BIRC5 protein levels in both cell types (Figure 5B). Importantly, combined treatment with iBAP-II and 5-FU led to enhanced apoptotic signaling compared with either treatment alone, and a similar increase in apoptotic signaling was observed following BAP1 knockdown in combination with 5-FU (Figure 5C and Figure S6). To further determine whether BIRC5 mediates the apoptotic effects of BAP1 depletion, we performed a rescue experiment by re-expressing BAP1 or BIRC5 in BAP1-depleted AGS cells. BAP1 knockdown reduced endogenous BIRC5 expression and increased the levels of cleaved caspase-3 and cleaved PARP. Notably, restoration of BAP1 or BIRC5 expression attenuated the induction of these apoptotic markers in BAP1-depleted cells (Supplementary Figure S7). These results further support BIRC5 as a functional downstream mediator of BAP1-dependent regulation of apoptosis in gastric cancer cells.

Cell viability assays further demonstrated that inhibition of BAP1 markedly increased the sensitivity of both parental and 5-FU resistant gastric cancer cells to 5-FU treatment (Figure 5D). Notably, in SNU620/5-FU cells, 5-FU alone did not significantly reduce viability relative to vehicle at day 3 (*p* = 0.728), confirming the resistant phenotype, whereas the combination of iBAP-II and 5-FU produced a significant reduction relative to both vehicle and 5-FU alone (both *p* < 0.001, Tukey’s HSD). A concordant pattern was observed for apoptosis (Figure 5E), in which 5-FU alone did not differ from vehicle (13.8% versus 13.0%, *p* = 0.964) whereas the combination increased apoptosis to 82.3% (*p* < 0.001). Flow cytometry analysis confirmed that the combination of iBAP-II and 5-FU induced significantly higher levels of apoptosis (Figure 5E). To ask whether this relationship is also apparent in patients, we returned to the pooled microarray cohort and stratified by adjuvant treatment. Consistent with BIRC5 acting as the effector of 5-FU resistance downstream of BAP1, BIRC5 expression was not associated with overall survival in the cohort as a whole (HR 0.88, 95% CI 0.74–1.04, *p* = 0.144) or among patients treated with surgery alone (HR 0.81, 95% CI 0.60–1.07, *p* = 0.141), but high BIRC5 expression was strongly associated with shorter survival among patients who received 5-FU-based adjuvant chemotherapy (n = 157; HR 1.73, 95% CI 1.22–2.45, log-rank *p* = 0.0019; median overall survival 15.2 versus 8.1 months; test for interaction *p* = 0.001). BAP1 itself showed no treatment-specific effect (interaction *p* = 0.74), as expected if its influence on 5-FU response is mediated through BIRC5 (Supplementary Figure S9 and Table S2C). Collectively, these findings suggest that targeting BAP1 can sensitize 5-FU resistant gastric cancer cells to 5-FU, and that the level of BIRC5 may be associated with differential outcomes following 5-FU-based treatment.

## 3. Discussion

In the present study, we identified BAP1 as an important regulator of gastric cancer progression and chemoresistance. Our results demonstrate that BAP1 is highly expressed in gastric cancer tissues and is associated with poor clinical prognosis. Functional studies further revealed that BAP1 promotes tumorigenic properties of gastric cancer cells, including proliferation, migration, and invasion. At the molecular level, we showed that BAP1 stabilizes the anti-apoptotic protein BIRC5 through deubiquitination activity. BIRC5 is a well-known inhibitor of apoptosis that suppresses caspase activation and enhances cancer cell survival. Overexpression of BIRC5 has been widely reported in multiple cancers and has been linked to tumor aggressiveness and resistance to anticancer therapies [20,21,22]. Our findings indicate that BAP1 interacts with BIRC5 and prevents its ubiquitin-mediated degradation, thereby maintaining high levels of BIRC5 protein in gastric cancer cells.

Although the regulatory relationship between BAP1 and BIRC5 has been reported in other contexts [15], our study highlights its functional significance in gastric cancer progression and, importantly, in chemoresistance to 5-FU. We found that BAP1 and BIRC5 expression levels were markedly elevated in 5-FU resistant SNU620 than normal SNU620 cells. It is noteworthy that BAP1 has been well-characterized as a tumor suppressor in mesothelioma and uveal melanoma, where biallelic inactivation drives oncogenesis [11]. Our finding that BAP1 promotes gastric cancer progression may initially appear contradictory; however, context-dependent dual roles of chromatin-modifying enzymes are increasingly recognized in cancer biology. In gastric cancer, which encompasses molecularly distinct subtypes including EBV-associated and chromosomally unstable tumors, the oncogenic function of BAP1 may reflect its deubiquitinase activity toward specific substrates such as BIRC5 that promote tumor cell survival. Whether elevated BAP1 and BIRC5 expression is a cause or a consequence of acquired 5-FU resistance remains to be fully elucidated. Future studies overexpressing BAP1 in chemosensitive parental cells and assessing changes in 5-FU sensitivity would clarify the directionality of this relationship. Importantly, pharmacological inhibition of BAP1 using the selective inhibitor iBAP-II significantly enhanced the sensitivity of gastric cancer cells to 5-FU treatment. Interestingly, iBAP-II treatment not only inhibited BAP1 enzymatic activity but also reduced BAP1 protein levels themselves (Figure 5B), suggesting that BAP1 may regulate its own stability through auto-deubiquitination—a positive feedback mechanism that could amplify the therapeutic effects of BAP1 inhibition. Inhibition of BAP1 resulted in reduced BIRC5 protein levels and activation of apoptotic signaling pathways, as evidenced by increased cleavage of caspase-3 and PARP. These findings suggest that targeting BAP1 may restore apoptotic responses and sensitize 5-FU resistant gastric cancer cells to 5-FU.

Several strategies for enhancing 5-FU chemosensitivity have also been reported in colorectal cancer. Deoxyelephantopin enhanced the response to 5-FU by promoting apoptosis through the miR-205/BCL2 axis [23], whereas HMBOX1 was reported to reverse autophagy-mediated 5-FU resistance by promoting HACE1-dependent ubiquitination and degradation of ATG5 [24]. More recently, Bacteroides fragilis was shown to promote resistance to 5-FU-based chemotherapy through Notch1-dependent induction of epithelial–mesenchymal transition and stemness, while targeting this pathway restored chemosensitivity [25]. Although these findings were obtained in colorectal cancer models and cannot be directly extrapolated to gastric cancer, they collectively illustrate that 5-FU responsiveness can be modulated by targeting diverse mechanisms that promote tumor cell survival. In this context, our findings suggest that regulation of BIRC5 protein stability by BAP1 may represent an additional mechanism influencing the response of gastric cancer cells to 5-FU.

Recent studies have increasingly highlighted the therapeutic potential of targeting the ubiquitin proteasome system in cancer treatment [26,27]. While proteasome inhibitors have been successfully used in hematologic malignancies [28], targeting specific deubiquitinases may offer a more selective approach for modulating oncogenic signaling pathways [29]. Our results support the idea that BAP1 represents a promising therapeutic target in gastric cancer, particularly in the context of chemotherapy resistance.

Nevertheless, this study has several limitations. First, the therapeutic effect of BAP1 inhibition in combination with 5-FU was evaluated primarily using in vitro gastric cancer cell models. Further studies using 5-FU resistant gastric cancer xenograft models are warranted to determine whether the chemosensitizing effect of BAP1 inhibition observed in vitro can be reproduced in vivo and to establish its potential translational relevance. In addition, the clinical relevance of BAP1 inhibition in combination with 5-FU-based chemotherapy should be evaluated in future translational studies.

In conclusion, this study establishes BAP1 as a novel oncogenic deubiquitinase in gastric cancer that promotes tumor progression and 5-FU chemoresistance through stabilization of the anti-apoptotic protein BIRC5. Pharmacological inhibition of BAP1 using iBAP-II effectively disrupted this axis, restoring apoptotic sensitivity and enhancing the efficacy of 5-FU, particularly in resistant cells. Future investigations should explore whether BAP1 expression levels can serve as a predictive biomarker for fluoropyrimidine responsiveness in gastric cancer patients, and whether BAP1 inhibition synergizes with other standard-of-care regimens such as oxaliplatin-based combinations or immune checkpoint inhibitors. Comprehensive elucidation of the upstream regulators and full spectrum of epigenetic targets of BAP1 in gastric cancer will further advance our understanding of its oncogenic mechanism and broaden its therapeutic potential.

## 4. Materials and Methods

### 4.1. Antibodies and Reagents

The antibodies used in this study are as follows: Anti-BAP1 (Santa Cruz Biotechnology, Dallas, TX, USA, sc-28383), Anti-β-Actin (Santa Cruz Biotechnology, sc-47778), Anti-Survivin (Cell signaling Technology, Danvers, MA, USA, 2803), Anti-PARP (Cell signaling Technology, 9542), Anti-Caspase-3 (Cell signaling Technology, 9662), Anti-MYC-tag (Cell signaling Technology, 2276), Anti-HA-tag (Cell signaling Technology, 3724), Goat anti-rabbit IgG HRP conjugated (CiteAb, Somerset, UK, ADI SAB 300 J), Goat anti-mouse IgG F(ab)2, polyclonal antibody (CiteAb, ADI SAB 100 J), Alexa Fluor™ 594 (Invitrogen, Carlsbad, CA, USA, A11012), Alexa Fluor™ 488 (Invitrogen, A11001), Anti-HA antibody–conjugated agarose beads (Sigma-Aldrich, St. Louis, MO, USA, A2095), Anti-c-Myc Agarose beads (Thermofisher, Waltham, MA, USA, 20169), cycloheximide (Sigma-Aldrich, 01810), Velcade (Sigma-Aldrich, 5.04314), iBAP-II (FOCUS, London, UK, 10-4038), 5-Fluorouracil (Sigma-Aldrich, F6627).

### 4.2. Cell Lines and Culture

AGS, MKN1, SNU484, SNU620, SNU620/5-FU cells were purchased from the Korean Cell Line Bank (KCLB, Seoul, Republic of Korea), and MKN74, HEK293T cells were purchased from the American Type Culture Collection (ATCC, Manassas, VA, USA). AGS, MKN74, and HEK293T cell lines were cultured in DMEM (Dulbecco’s Modified Eagle’s Medium, Corning Inc., Corning, NY, USA, 10-013-CV) supplemented with 10% FBS (Corning, 35-015-CV) and 1% Penicillin/Streptomycin (Gibco, Grand Island, NY, USA, 15140163). MKN1, SNU484, SNU620, and SNU620/5-FU cell lines were cultured in RPMI1640 (Corning, 10-040-CV) medium supplemented with 10% FBS and 1% Penicillin/Streptomycin. All cell lines were cultured in a 37 °C, 5% CO_2_ incubator. In addition, SNU620/5-FU cells were routinely maintained in medium containing 1 μM of 5-FU and cultured in drug-free medium for 2 days before experiments.

### 4.3. Western Blotting

Patient-derived gastric tissue was homogenized with scissors after adding RIPA buffer (50 mM Tris-HCl pH 7.5, 0.1% SDS, 1% Triton X-100, 150 mM NaCl, 0.5% Sodium deoxycholate, 2 mM EDTA) (iNtRON, Seongnam-si, Republic of Korea, BR002) with 1X Protease inhibitor (GenDEPOT, Barker, TX, USA, p3100-020) and reacted at 4 °C for 30 min. After centrifugation at 13,000 rpm for 30 min at 4 °C, protein extracts were obtained and heated at 98 °C for 7 min with β-Mercaptoethanol (Sigma-Aldrich). Protein concentration was determined by BCA assay kit (Pierce, Rockford, IL, USA, 23225) and ~40 µg of protein was loaded onto SDS-PAGE gels and transferred to PVDF membrane (Sigma-Aldrich, IPVH00010). Blocking was performed for 1 h at room temperature using 5% Skim milk (LPS, Daejeon, Republic of Korea, SKI500) in PBST solution, and the primary antibody was reacted at 4 °C for more than 12 h. After reacting the secondary antibody at room temperature for 1 h, the protein was detected using ECL (Thermofisher, 34579). The cells were lysed with SDS-lysis buffer (50 mM Tris pH 7.4, 150 mM NaCl, 1% SDS, 1 mM EDTA) using 23G needle syringe. The extracted protein concentration was determined by BCA assay kit, and appropriate amounts of protein were loaded onto SDS-PAGE gels for Western blot analysis.

### 4.4. Transfection

The plasmids (MYC-BAP1, HA-BIRC5, MYC-BAP1 WT, MYC-BAP1 C91A, and FLAG-UB) were generated using Gateway cloning technology (Invitrogen, 11791020).

Lipofectamine 3000 (Invitrogen, L3000150), P3000 reagent, and plasmid DNAs were mixed with OptiMEM (Gibco, 31985088) and incubated at room temperature for 15 min. The DNA-lipid complex was treated on 2–3 × 10^5^ cells per 6-well culture plates. After 48 h, the transfected cells were lysed with SDS-lysis buffer and performed Western blotting analysis. The small interfering RNAs (siRNAs) for human BAP1 were synthesized from Genolution and Bioneer. RNAiMAX (Invitrogen, 13778500), OptiMEM, and ~40 pmole of siRNAs were reacted at room temperature for 15 min for siRNA-lipid complex and then added to 2–3 × 10^5^ cells per 6-well culture plates. After 72 h, the transfection cells were lysed with SDS-lysis buffer and performed Western blotting analysis. The sequences targeting BAP1 were as follows:

siBAP1 #1(sense, 5′-CGACCUUCAGAGCAAAUGUUU-3′);

siBAP1 #2(sense, 5′-GAGUUCAUCUGCACCUUUAUU-3′).

### 4.5. Cell Viability

Cell proliferation analysis was performed using the CellTiter-Glo Luminescent Cell Viability Assay Kit (Promega, Madison, WI, USA, G9241). Cells were seeded into a 96-well white plate at a concentration of 1 × 10^3^ cells/100 µL and cultured in 37 °C, 5% CO_2_ incubator. After stabilization at room temperature for 30 min, 100 µL of Cell Titer-Glo reagent diluted with PBS was added to each well. The plate was mixed on a shaker at room temperature for 5 min maintaining protected from light. The luminescence intensity was measured using a GloMax 96 Microplate Luminometer (Promega, Madison, WI, USA). Cell viability was analyzed every 24 h for 3 days. Different concentrations of iBAP-II were used according to the experimental endpoint and duration of exposure. For short-term Western blotting and apoptosis experiments, cells were treated with 48 or 64 μM iBAP-II for 24 h to obtain measurable changes in BAP1/BIRC5 expression and apoptotic signaling. For the 3-day cell-viability assay, a lower concentration of 10 μM was used to minimize nonspecific cytotoxicity during prolonged exposure and to evaluate the chemosensitizing effect of iBAP-II in combination with 5-FU.

### 4.6. Colony Forming Assay

A total of 1 × 10^3^ cells were seeded per 6-well culture plate, and cultured in a 37 °C, 5% CO_2_ incubator for 1–2 weeks. Cells were fixed with 4% paraformaldehyde at room temperature for 20 min, stained with 0.5% crystal violet at room temperature for 5 min, and the number of colonies was counted.

### 4.7. Transwell Migration and Invasion

For the migration assay, 2 × 10^4^ cells in 200 µL of medium without FBS were seeded on the upper chamber of 24-well Transwell inserts (SPL, Pocheon-si, Republic of Korea) upper chamber, and lower chamber was filled with 500 µL of complete medium. After 24 h, upper chamber inserts were fixed with 4% paraformaldehyde at room temperature for 20 min, stained with 0.5% crystal violet at room temperature for 5 min, and the number of migrated cells was counted.

For the invasion assay, Matrigel (Corning) diluted in cold PBS was pre-coated on 24-well Transwell inserts upper chamber for 2 h in a 37 °C, 5% CO_2_ incubator. 2 × 10^4^ cells in 200 µL of medium without FBS were seeded on a 24-well Transwell inserts (SPL) upper chamber, and lower chamber was filled 500 µL of complete medium. After 24 h, upper chamber inserts were fixed with 4% paraformaldehyde at room temperature for 20 min, stained with 0.5% crystal violet at room temperature for 5 min, and counted.

### 4.8. Flow Cytometry

Flow cytometry analysis for apoptosis was performed using the Annexin V FITC Apoptosis Detection Kit (BD, San Jose, CA, USA, 556547). 5 × 10^5^ cells were washed with PBS, resuspended in 1X Binding Buffer (HEPES/NaOH (pH 7.4), 1.4 M NaCl, 25 mM CaCl_2_), and stained with PI and Annexin V at room temperature for 15 min while protected from light. Stained cells were analyzed using the Canto II Flow Cytometry system (BD). 10,000 events were acquired per sample and cellular debris was excluded based on forward scatter (FSC) and side scatter (SSC) characteristics, and single cells were selected for subsequent Annexin V/PI analysis. Annexin V−/PI− cells were considered viable, Annexin V+/PI− cells were classified as early apoptotic, and Annexin V+/PI+ cells were classified as late apoptotic/secondary necrotic. Total apoptotic cells were calculated as the sum of the early and late apoptotic populations.

### 4.9. Immunofluorescence

After washing the cells with PBS, cells were fixed with 4% paraformaldehyde at room temperature for 20 min. And added 0.1% Triton X-100 at room temperature for 5 min. Blocking was performed for 1 h at room temperature using 3% bovine serum albumin (BSA) in PBS, and the primary antibody was reacted at 4 °C for more than 12 h. After reacting the secondary antibody at room temperature for 1 h, DAPI solution was added at room temperature for 5 min while protected from light. Cells were fixed to glass slides using mounting reagent (Invitrogen, P36930), and imaged using a confocal microscope (Carl Zeiss AG, Oberkochen, Germany, LSM-780).

### 4.10. Immunohistochemistry

Patient tissue slides were deparaffinized using xylene and hydrated by using ethanol (90%, 80%, 70%). Antigen retrieval was performed using 1× Citrate reagent (Biosesang, Yongin-si, Republic of Korea CR2072-100), and the peroxidase in the tissue was inhibited by reacting with 3% hydrogen peroxide at room temperature for 20 min. Blocking was performed with 1% BSA at room temperature for 1 h, reacted with α-BAP1 antibody (1:100) at 4 °C for more than 12 h. The secondary antibody was reacted at room temperature for 1 h and stained with DAB (Agilent, Santa Clara, CA, USA, K3468) and Hematoxylin (Abcam, Waltham, MA, USA, ab220365). After dehydration with ethanol and xylene, the slides were mounted and observed under a microscope.

### 4.11. Immunoprecipitation

Cells were harvested and homogenized using 22G syringes after adding MCLB buffer (50 mM Tris pH 7.4, 150 mM NaCl, 0.5% Nonidet P-40, 1 mM EDTA) with 1× Protease inhibitor (GenDEPOT, p3100-020) and reacted at 4 °C for 30 min. After centrifugation at 13,000 rpm for 30 min 4 °C, the supernatant was incubated with α-HA antibody-conjugated agarose beads (Sigma-Aldrich) or α-MYC antibody-conjugated agarose beads (Sigma-Aldrich) at 4 °C for at least 12 h. After washing with MCLB buffer, Western blotting was performed.

### 4.12. Cycloheximide Chasing Assay

Cells were transfected with the indicated siRNAs or expression plasmids. Following transfection, cells were treated with cycloheximide at a final concentration of 50 μg/mL and harvested at the indicated time points. Lysates were prepared and subjected to Western blot analysis using indicated antibodies. β-actin was used as a loading control. BIRC5 protein levels were quantified by densitometric analysis, normalized to β-actin, and expressed relative to the corresponding level at 0 h.

### 4.13. Ubiquitination Assay

The 293T cells were transfected with the indicated expression plasmids encoding of MYC-BAP1, HA-BIRC5, and FLAG-Ubiquitin. After 48 h of transfection, cells were treated with the Velcade at 50 ng/mL for 6 h before collection. Cells were lysed, and BIRC5 was immunoprecipitated using the indicated antibody. The immunoprecipitated proteins were subsequently subjected to Western blot analysis, and ubiquitinated BIRC5 was detected using an anti-FLAG antibody. Expression of the indicated proteins in whole-cell lysates was confirmed by Western blotting.

### 4.14. Survival Analysis

Overall survival in relation to BAP1 and BIRC5 expression was analysed using the Kaplan–Meier plotter, a resource based on pooled gastric cancer microarray cohorts (*n* = 875). BAP1 was assessed with probe 201419_at and BIRC5 with probe 202095_s_at. For Figure 1C the cut-point was selected using the tool’s optimization function; all other analyses used a pre-specified median split. Subgroups were defined using the clinicopathological and treatment annotation provided by the resource. Groups were compared by the log-rank test, and hazard ratios with 95% confidence intervals are reported. Differences between treatment subgroups were assessed by a Wald test on the difference in log hazard ratios.

All tests were two-sided, and *p* < 0.05 was considered statistically significant. Significance is indicated as * *p* < 0.05, ** *p* < 0.01 and *** *p* < 0.001; ns denotes not significant. Analyses were performed using GraphPad Prism (version 10; GraphPad Software, San Diego, CA, USA) and Python 3.11 with the SciPy (v1.14) and statsmodels (v0.14) libraries.

### 4.15. Statistical Analysis

All experiments were performed as at least three independent biological replicates, each initiated from a separate cell passage and transfection or treatment. The exact number of independent replicates (*n*) for every experiment is stated in the corresponding figure legend and is summarised in Supplementary Table S1. Data are presented as mean ± standard deviation (SD), and individual data points are shown where applicable.

Time-course cell viability data (CellTiter-Glo) were analysed by two-way ANOVA with treatment and time as factors. A repeated-measures model was applied to datasets in which all replicates were complete, and an ordinary two-way ANOVA was used where isolated measurements were missing, because repeated-measures models exclude incomplete cases. When the treatment × time interaction was significant, simple effects were examined by one-way ANOVA at each time point, followed by Dunnett’s post hoc test when all groups were compared with a single control (siRNA experiments) or Tukey’s honestly significant difference (HSD) test when all pairwise comparisons were of interest (combination-treatment experiments).

Single-time-point experiments with three or more groups (colony formation, transwell migration and invasion, and flow cytometric apoptosis assays) were analysed by one-way ANOVA followed by Dunnett’s or Tukey’s HSD post hoc test, as appropriate. Experiments comparing only two groups were analysed by a two-sided Student’s *t*-test, for which no correction for multiple comparisons is required. Western blot, immunoprecipitation, immunofluorescence and cycloheximide chase experiments are shown as representative images of three independent experiments.

## Data Availability

The data are contained within the article and the supplementary material.

## Supplementary Materials

The following supporting information can be downloaded at: https://www.mdpi.com/article/10.3390/ph19101593/s1, Figure S1. The protein level of BAP1 in gastric cancer patients’ normal and tumor tissues. Supplementary Figure S2. BAP1 knockdown inhibits proliferation of SNU620 and SNU620/5-FU cells. Supplementary Figure S3. BAP1 regulates BIRC5 protein stability. Supplementary Figure S4. BAP1 regulates BIRC5 protein stability via proteasomal degradation. Supplementary Figure S5. BAP1 knockdown induces apoptosis by reducing BIRC5 expression in SNU620 and SNU620/5-FU cells. Supplementary Figure S6. Depletion of BAP1 enhances 5-FU-induced apoptotic signaling. Supplementary Figure S7. Restoration of BAP1 and BIRC5 attenuates 5-FU-induced apoptosis following BAP1 depletion. Supplementary Figure S8. Association of BAP1 expression with overall survival within clinicopathological strata. Supplementary Figure S9. Association of BAP1 and BIRC5 expression with overall survival by adjuvant treatment. Supplementary Figure S10. Validation of 5-FU resistance in SNU620/5-FU cells. Table S1A. Two-way ANOVA of time-course cell viability experiments. Table S1B. One-way ANOVA with post hoc multiple-comparison tests. Table S1C. Number of independent biological replicates per experiment. Table S2A. Association of BAP1 and BIRC5 expression with overall survival in gastric cancer. Supplementary Table S2B. BAP1 (201419_at) and overall survival, stratified by clinicopathological variable. Table S2C. BAP1 and BIRC5 and overall survival, stratified by adjuvant treatment.
