# Supplementary material for: Inhibition of BAP1 Sensitizes Gastric Cancer Cells to 5-Fluorouracil by Promoting BIRC5 Degradation
Source: Pharmaceuticals (Basel). 2026 Oct 8;19(10):1593. doi: 10.3390/ph19101593 (PMC13708599; doi:10.3390/ph19101593)
Supplement: Supplementary file 1 [file pharmaceuticals-19-01593-s001.zip › pharmaceuticals-4553581-supplementary.pdf]

Inhibition of BAP1 Sensitizes Gastric Cancer Cells to 5-Fluorouracil by Promoting BIRC5 Degradation

Supplementary Figure S1

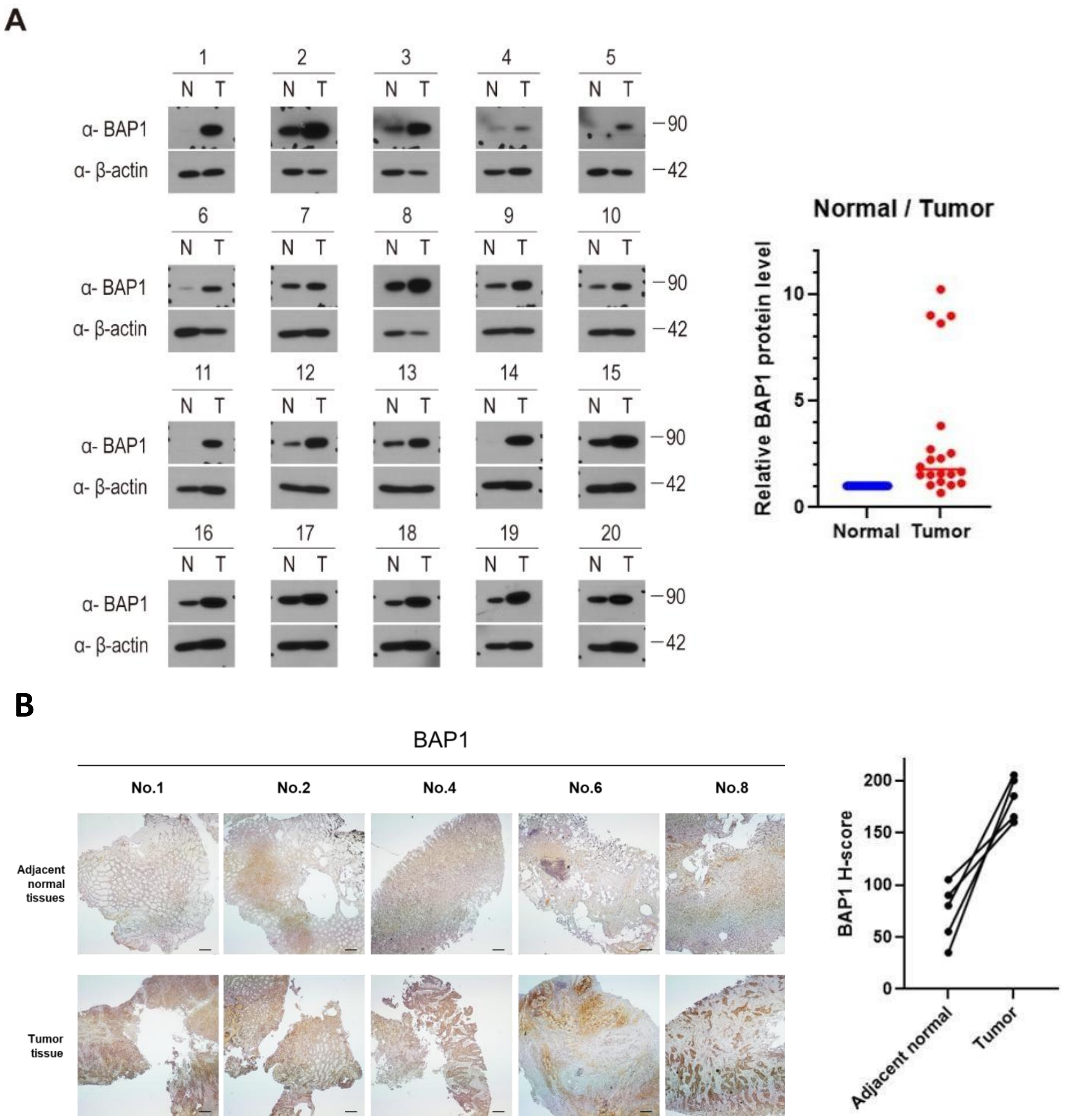

Supplementary Figure S1. The protein level of BAP1 in gastric cancer patients' normal and tumor tissues. (A) Western blot results in 20 paired adjacent normal gastric tissues (N) and tumor tissues (T). BAP1 protein levels were normalized to β-actin, and tumor expression was calculated relative to the corresponding adjacent normal tissue. Quantitative analysis of all 20 paired samples showed significantly increased BAP1 expression in gastric tumor tissues (median relative tumor expression = 1.786; n = 20; $P < 0.0001$, Wilcoxon signed-rank test). **(B)** Immunohistochemical staining of BAP1 in gastric tumor and adjacent normal tissues. IHC analysis was performed in five paired specimens (patients #1, #2, #4, #6, and #8), and BAP1 immunoreactivity was semi-quantitatively evaluated using an H-score based on staining intensity and the proportion of positively stained cells. Quantitative analysis showed significantly higher BAP1 H-scores in gastric tumor tissues than in paired adjacent normal tissues (n = 5; P = 0.0050, paired t-test).

Supplementary Figure S2

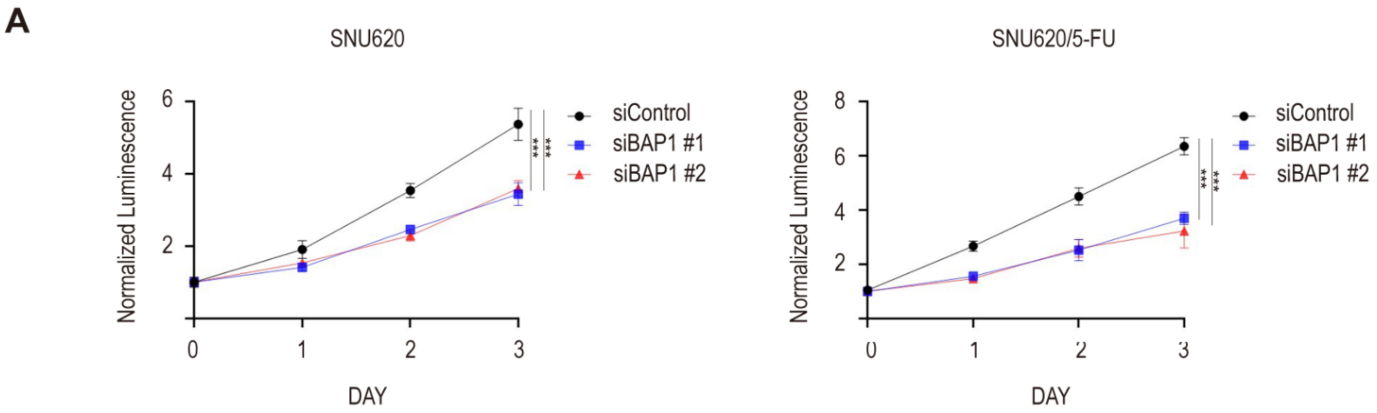

Supplementary Figure S2. BAP1 knockdown inhibits proliferation of SNU620 and SNU620/5-FU cells. (A) Effects of BAP1 knockdown on the viability of SNU620 and SNU620/5-FU cells. Cell viability was monitored for 3 days using a CellTiter-Glo luminescent assay (normalized to control siRNA at day 0; *** $p < 0.001$).

Supplementary Figure S3

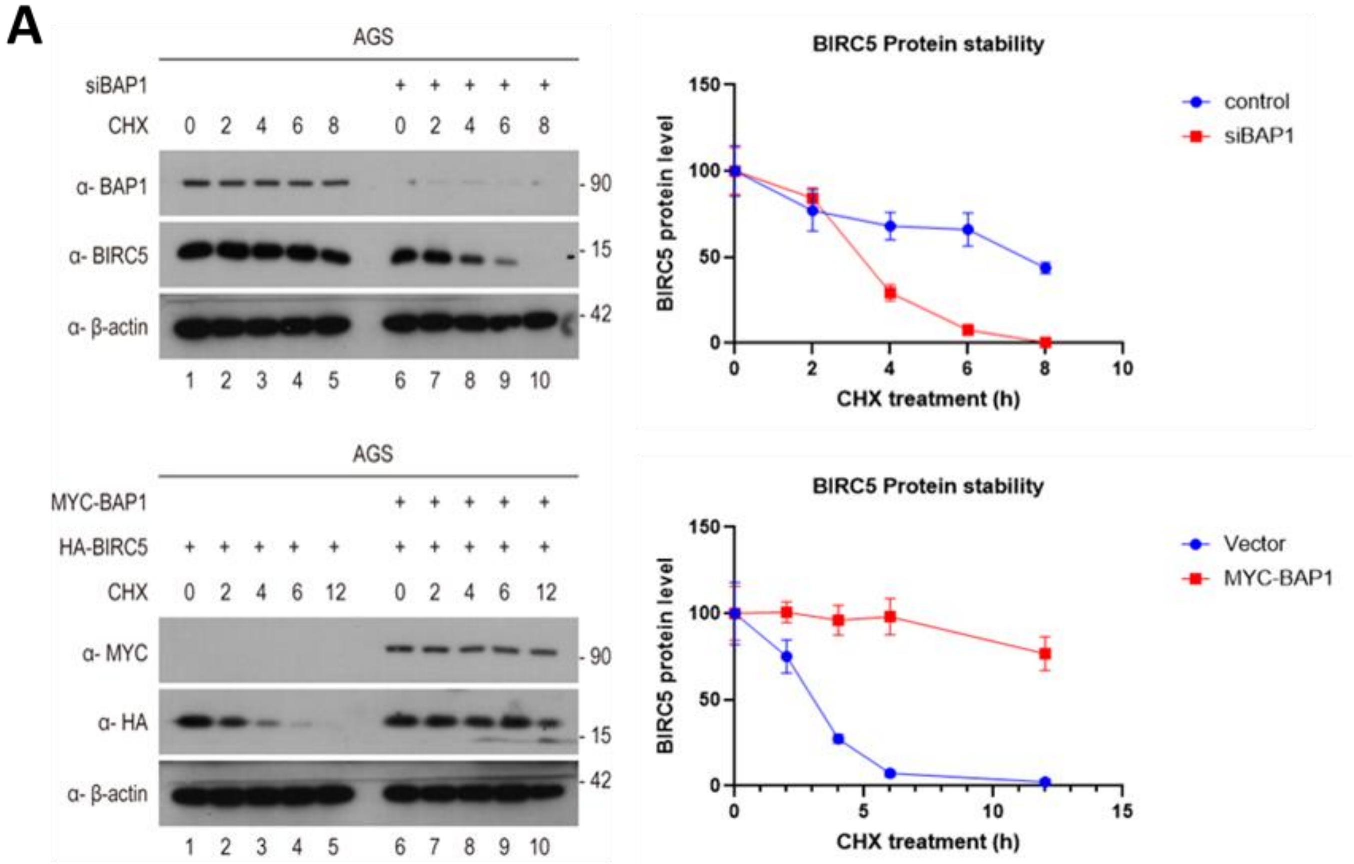

Supplementary Figure S3. BAP1 regulates BIRC5 protein stability. (A) (upper lane) AGS cells were transfected with control or BAP1 siRNA and subsequently treated with cycloheximide for the indicated times. BAP1 and BIRC5 protein levels were analyzed by western blotting. BIRC5 levels were normalized to β-actin and expressed relative to the corresponding 0h value. **(lower lane)** AGS cells were co-transfected with HA-BIRC5 and either empty vector or MYC-BAP1 and treated with CHX for the indicated times. BIRC5 protein levels were quantified relative to β-actin and normalized to the corresponding 0h value.

Supplementary Figure S4

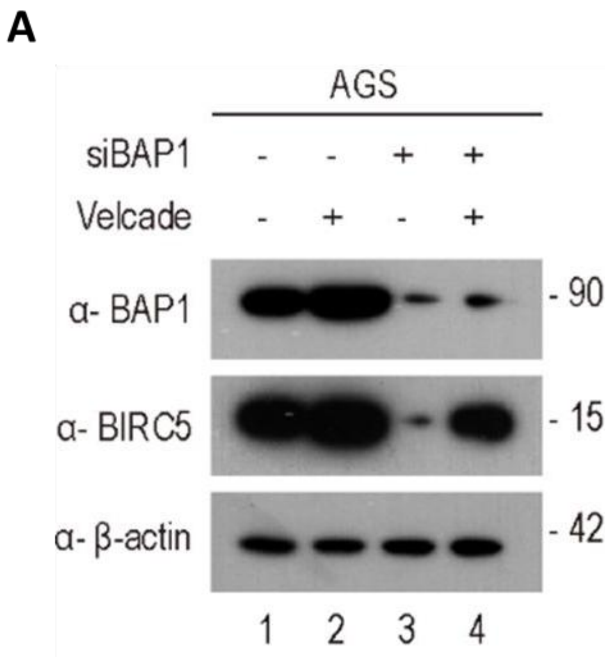

Supplementary Figure S4. BAP1 regulates BIRC5 protein stability via proteasomal degradation. (A) AGS cells transfected with control or BAP1 siRNA were treated with the proteasome inhibitor Velcade as indicated. BAP1 and BIRC5 protein levels were analyzed by western blotting, with β-actin used as a loading control.

Supplementary Figure S5

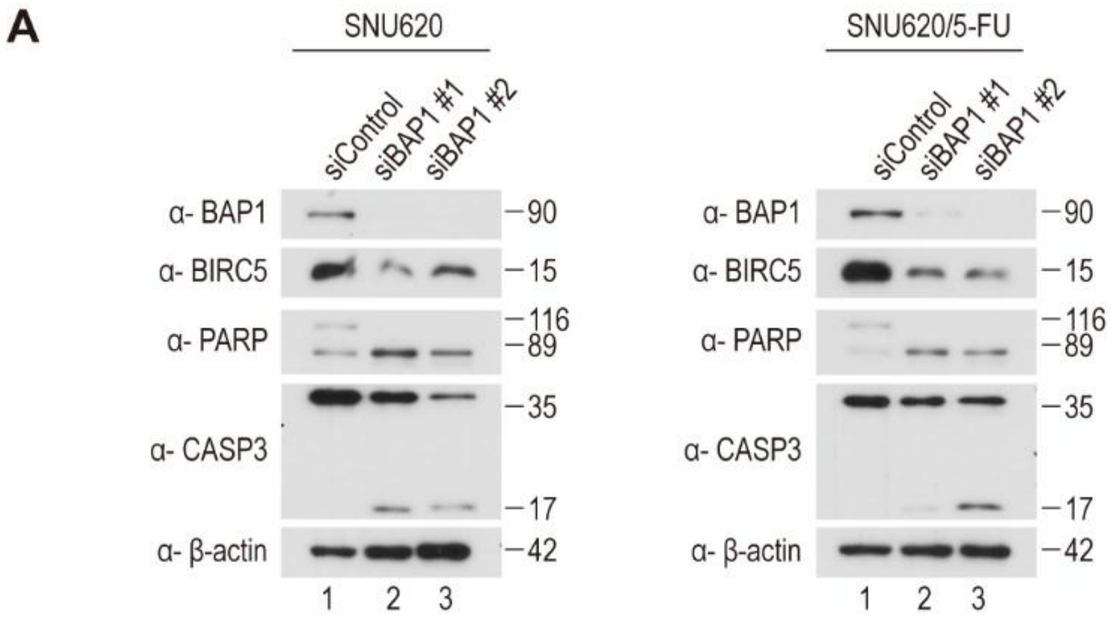

Supplementary Figure S5. BAP1 knockdown induces apoptosis by reducing BIRC5 expression in SNU620 and SNU620/5-FU cells. (A) Western blot analysis of apoptotic markers following BAP1 knockdown in SNU620 and SNU620/5-FU cells. After 72 h incubation, the cells were lysed and immunoblotted with the indicated antibodies.

Supplementary Figure S6

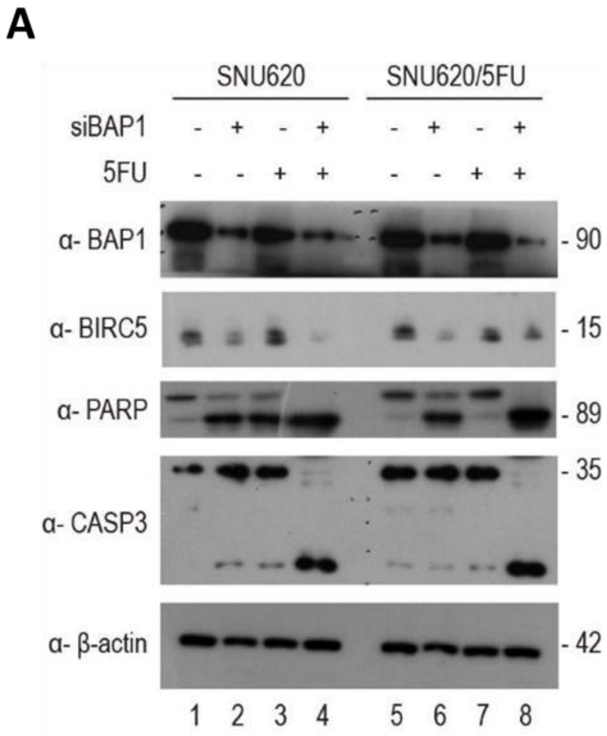

Supplementary Figure S6. Depletion of BAP1 enhances 5-FU-induced apoptotic signaling. (A) SNU620 and SNU620/5-FU cells were transfected with control or BAP1 siRNA and subsequently treated with 5-FU as indicated. Protein levels of BAP1, BIRC5, PARP, and caspase-3 were analyzed by western blotting, with β- actin used as a loading control.

Supplementary Figure S7

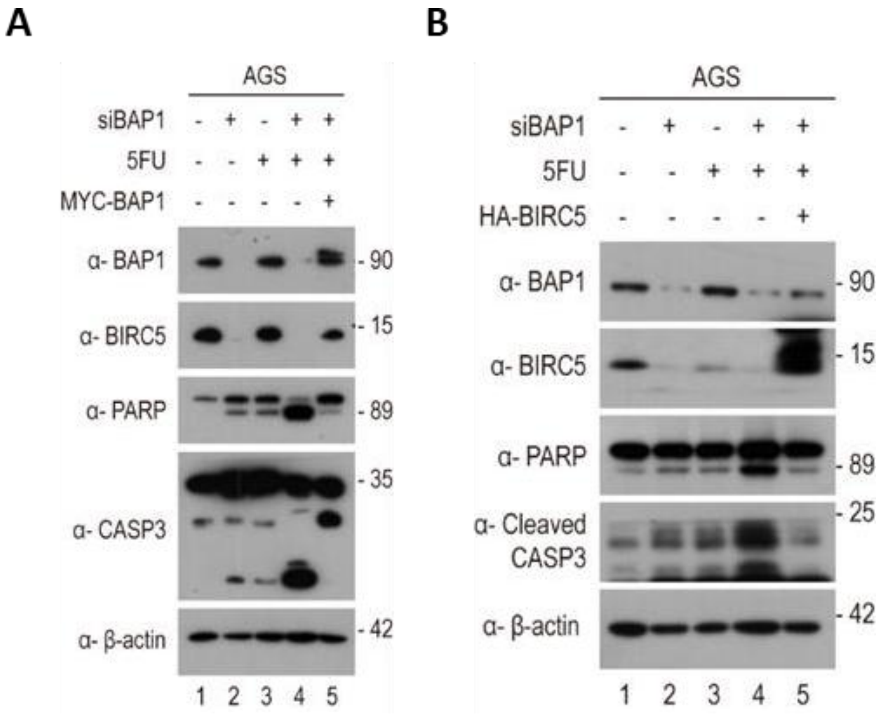

Supplementary Figure S7. Restoration of BAP1 and BIRC5 attenuates 5-FU-induced apoptosis following BAP1 depletion. (A) AGS cells were transfected with BAP1 siRNA, followed by MYC-BAP1 re-expression and 5-FU treatment as indicated. Protein levels of BAP1, BIRC5, PARP, and caspase-3 were analyzed by western blotting, with β-actin used as a loading control. **(B)** AGS cells were transfected with control or BAP1 siRNA, followed by re-expression of HA-BIRC5 as indicated. Protein levels of BAP1, BIRC5, PARP, and caspase3 were analyzed by western blotting, with β-actin used as a loading control.

Supplementary Figure S8

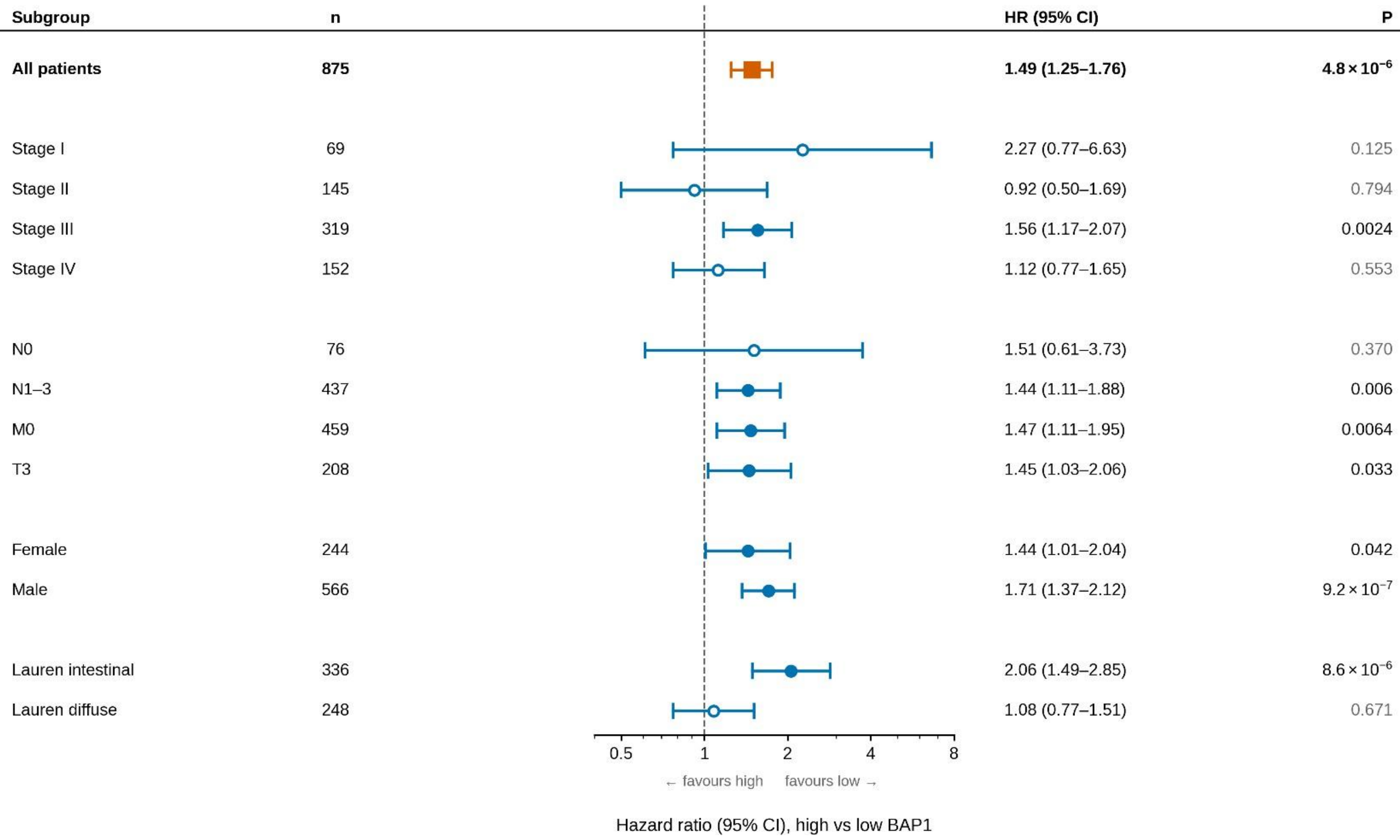

Supplementary Figure S8. Association of BAP1 expression with overall survival within clinicopathological strata. Overall survival according to BAP1 expression (probe 201419_at) in a pooled gastric cancer microarray cohort (Kaplan–Meier plotter). Patients were dichotomised at the median within each stratum. Squares and circles indicate hazard ratios and horizontal lines the 95% confidence intervals; filled symbols denote strata in which the confidence interval excludes 1. n, number of patients with the relevant annotation. P values are from the log-rank test.

Supplementary Figure S9

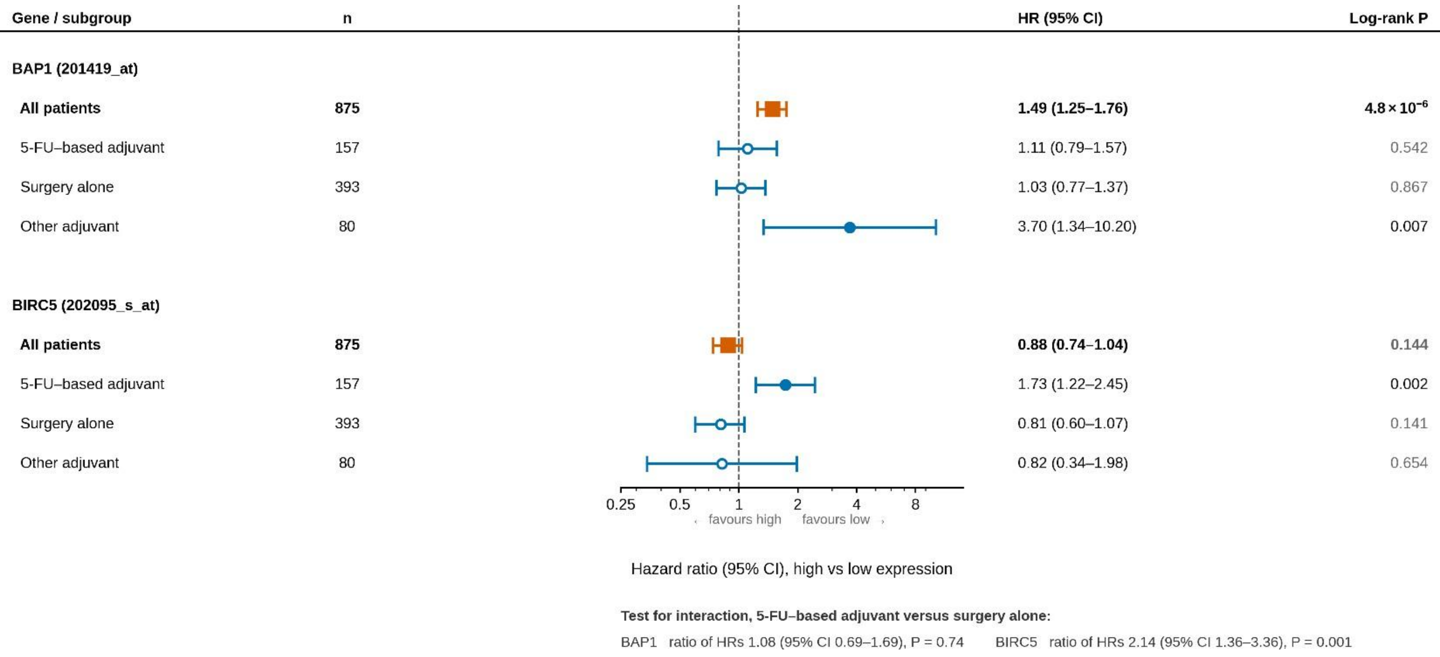

Supplementary Figure S9. Association of BAP1 and BIRC5 expression with overall survival by adjuvant treatment. Overall survival according to BAP1 (probe 201419_at) and BIRC5 (probe 202095_s_at) expression in a pooled gastric cancer microarray cohort (Kaplan–Meier plotter), stratified by adjuvant treatment. Patients were dichotomised at the median within each subgroup. Squares and circles indicate hazard ratios and horizontal lines the 95% confidence intervals; filled symbols denote subgroups in which the confidence interval excludes 1. P values are from the log-rank test. The test for interaction compares the log hazard ratios of the 5-FU-based adjuvant and surgery-alone subgroups by a Wald test on their difference.

Supplementary Figure S10

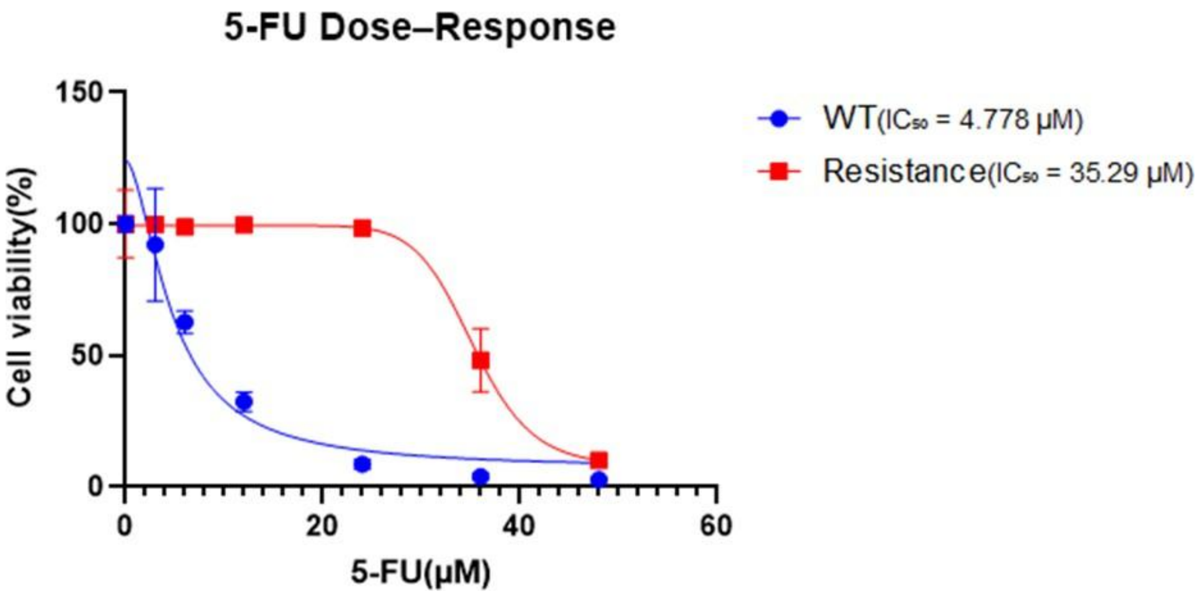

Supplementary Figure S10. Validation of 5-FU resistance in SNU620/5-FU cells. Dose–response curves of parental SNU620 and SNU620/5-FU cells following treatment with increasing concentrations of 5-FU. Cell viability was assessed after 3 days using a CellTiter-Glo luminescent assay and normalized to the corresponding untreated control for each cell line.

Supplementary Table S1A

Supplementary Table S1A. Two-way ANOVA of time-course cell viability experiments

Repeated-measures ANOVA used where all replicates were complete; ordinary two-way ANOVA where some replicates were missing. n = 3–5 independent biological experiments.

Figure	Cell line / experiment	Model	Effect	F value	df (num, den)	P value
Figure 2B	AGS (siBAP1)	Two-way RM ANOVA	Treatment	553	2, 8	<0.001
Figure 2B	AGS (siBAP1)	Two-way RM ANOVA	Time	297.4	3, 12	<0.001
Figure 2B	AGS (siBAP1)	Two-way RM ANOVA	Treatment × Time	83.4	6, 24	<0.001
Figure 2B	SNU484 (siBAP1)	Two-way RM ANOVA	Treatment	95.7	2, 8	<0.001
Figure 2B	SNU484 (siBAP1)	Two-way RM ANOVA	Time	1114.6	3, 12	<0.001
Figure 2B	SNU484 (siBAP1)	Two-way RM ANOVA	Treatment × Time	62.3	6, 24	<0.001
Suppl. Fig	SNU620 (siBAP1)	Two-way RM ANOVA	Treatment	52.9	2, 8	<0.001
Suppl. Fig	SNU620 (siBAP1)	Two-way RM ANOVA	Time	1232.8	3, 12	<0.001
Suppl. Fig	SNU620 (siBAP1)	Two-way RM ANOVA	Treatment × Time	31	6, 24	<0.001
Suppl. Fig	SNU620/5FU (siBAP1)	Two-way RM ANOVA	Treatment	159.1	2, 8	<0.001
Suppl. Fig	SNU620/5FU (siBAP1)	Two-way RM ANOVA	Time	526	3, 12	<0.001
Suppl. Fig	SNU620/5FU (siBAP1)	Two-way RM ANOVA	Treatment × Time	32.7	6, 24	<0.001
Figure 5D	SNU620 (iBAP-II/5FU)	Two-way ANOVA	Treatment	62.4	3, 52	<0.001
Figure 5D	SNU620 (iBAP-II/5FU)	Two-way ANOVA	Time	622.5	3, 52	<0.001
Figure 5D	SNU620 (iBAP-II/5FU)	Two-way ANOVA	Treatment × Time	26.9	9, 52	<0.001
Figure 5D	SNU620/5FU (iBAP-II/5FU)	Two-way ANOVA	Treatment	83.3	3, 56	<0.001
Figure 5D	SNU620/5FU (iBAP-II/5FU)	Two-way ANOVA	Time	660.2	3, 56	<0.001
Figure 5D	SNU620/5FU (iBAP-II/5FU)	Two-way ANOVA	Treatment × Time	28.8	9, 56	<0.001

Supplementary Table S1B

Supplementary Table S1B. One-way ANOVA with post hoc multiple-comparison tests

*Dunnett's test used when all groups were compared with a single control; Tukey's HSD when all pairwise comparisons were of interest. Significance: *P<0.05, **P<0.01, ***P<0.001; ns, not significant.*

Figure	Assay	Cell line	Timepoint	Comparison	n (independent experiments)	Test	Group means / statistic	P value	Significance
Figure 2B	Cell viability (CellTiter-Glo)	AGS (siBAP1)	Day 1	Overall (one-way ANOVA)	5	One-way ANOVA	F(2,12) = 10.5	0.002	**
Figure 2B	Cell viability (CellTiter-Glo)	AGS (siBAP1)	Day 1	Con vs siBAP1#1	5 vs 5	Dunnett's post hoc	2.64 vs 2.15	0.002	**
Figure 2B	Cell viability (CellTiter-Glo)	AGS (siBAP1)	Day 1	Con vs siBAP1#2	5 vs 5	Dunnett's post hoc	2.64 vs 2.59	0.842	ns
Figure 2B	Cell viability (CellTiter-Glo)	AGS (siBAP1)	Day 2	Overall (one-way ANOVA)	5	One-way ANOVA	F(2,12) = 6.5	0.012	*
Figure 2B	Cell viability (CellTiter-Glo)	AGS (siBAP1)	Day 2	Con vs siBAP1#1	5 vs 5	Dunnett's post hoc	4.11 vs 3.17	0.007	**
Figure 2B	Cell viability (CellTiter-Glo)	AGS (siBAP1)	Day 2	Con vs siBAP1#2	5 vs 5	Dunnett's post hoc	4.11 vs 3.64	0.159	ns
Figure 2B	Cell viability (CellTiter-Glo)	AGS (siBAP1)	Day 3	Overall (one-way ANOVA)	5	One-way ANOVA	F(2,12) = 62.4	<0.001	***
Figure 2B	Cell viability (CellTiter-Glo)	AGS (siBAP1)	Day 3	Con vs siBAP1#1	5 vs 5	Dunnett's post hoc	6.65 vs 3.35	<0.001	***
Figure 2B	Cell viability (CellTiter-Glo)	AGS (siBAP1)	Day 3	Con vs siBAP1#2	5 vs 5	Dunnett's post hoc	6.65 vs 4.29	<0.001	***
Figure 2B	Cell viability (CellTiter-Glo)	SNU484 (siBAP1)	Day 1	Overall (one-way ANOVA)	5	One-way ANOVA	F(2,12) = 76.2	<0.001	***
Figure 2B	Cell viability (CellTiter-Glo)	SNU484 (siBAP1)	Day 1	Con vs siBAP1#1	5 vs 5	Dunnett's post hoc	1.95 vs 1.36	<0.001	***
Figure 2B	Cell viability (CellTiter-Glo)	SNU484 (siBAP1)	Day 1	Con vs siBAP1#2	5 vs 5	Dunnett's post hoc	1.95 vs 1.47	<0.001	***
Figure 2B	Cell viability (CellTiter-Glo)	SNU484 (siBAP1)	Day 2	Overall (one-way ANOVA)	5	One-way ANOVA	F(2,12) = 136.8	<0.001	***
Figure 2B	Cell viability (CellTiter-Glo)	SNU484 (siBAP1)	Day 2	Con vs siBAP1#1	5 vs 5	Dunnett's post hoc	3.20 vs 1.92	<0.001	***
Figure 2B	Cell viability (CellTiter-Glo)	SNU484 (siBAP1)	Day 2	Con vs siBAP1#2	5 vs 5	Dunnett's post hoc	3.20 vs 1.99	<0.001	***
Figure 2B	Cell viability (CellTiter-Glo)	SNU484 (siBAP1)	Day 3	Overall (one-way ANOVA)	5	One-way ANOVA	F(2,12) = 87.8	<0.001	***
Figure 2B	Cell viability (CellTiter-Glo)	SNU484 (siBAP1)	Day 3	Con vs siBAP1#1	5 vs 5	Dunnett's post hoc	5.39 vs 2.70	<0.001	***
Figure 2B	Cell viability (CellTiter-Glo)	SNU484 (siBAP1)	Day 3	Con vs siBAP1#2	5 vs 5	Dunnett's post hoc	5.39 vs 3.15	<0.001	***
Suppl. Fig	Cell viability (CellTiter-Glo)	SNU620 (siBAP1)	Day 1	Overall (one-way ANOVA)	5	One-way ANOVA	F(2,12) = 13.0	<0.001	***
Suppl. Fig	Cell viability (CellTiter-Glo)	SNU620 (siBAP1)	Day 1	Con vs siBAP1#1	5 vs 5	Dunnett's post hoc	1.91 vs 1.41	<0.001	***
Suppl. Fig	Cell viability (CellTiter-Glo)	SNU620 (siBAP1)	Day 1	Con vs siBAP1#2	5 vs 5	Dunnett's post hoc	1.91 vs 1.54	0.006	**
Suppl. Fig	Cell viability (CellTiter-Glo)	SNU620 (siBAP1)	Day 2	Overall (one-way ANOVA)	5	One-way ANOVA	F(2,12) = 95.9	<0.001	***
Suppl. Fig	Cell viability (CellTiter-Glo)	SNU620 (siBAP1)	Day 2	Con vs siBAP1#1	5 vs 5	Dunnett's post hoc	3.54 vs 2.46	<0.001	***
Suppl. Fig	Cell viability (CellTiter-Glo)	SNU620 (siBAP1)	Day 2	Con vs siBAP1#2	5 vs 5	Dunnett's post hoc	3.54 vs 2.29	<0.001	***
Suppl. Fig	Cell viability (CellTiter-Glo)	SNU620 (siBAP1)	Day 3	Overall (one-way ANOVA)	5	One-way ANOVA	F(2,12) = 50.2	<0.001	***
Suppl. Fig	Cell viability (CellTiter-Glo)	SNU620 (siBAP1)	Day 3	Con vs siBAP1#1	5 vs 5	Dunnett's post hoc	5.38 vs 3.44	<0.001	***
Suppl. Fig	Cell viability (CellTiter-Glo)	SNU620 (siBAP1)	Day 3	Con vs siBAP1#2	5 vs 5	Dunnett's post hoc	5.38 vs 3.60	<0.001	***
Suppl. Fig	Cell viability (CellTiter-Glo)	SNU620/5FU (siBAP1)	Day 1	Overall (one-way ANOVA)	5	One-way ANOVA	F(2,12) = 129.7	<0.001	***
Suppl. Fig	Cell viability (CellTiter-Glo)	SNU620/5FU (siBAP1)	Day 1	Con vs siBAP1#1	5 vs 5	Dunnett's post hoc	2.67 vs 1.56	<0.001	***
Suppl. Fig	Cell viability (CellTiter-Glo)	SNU620/5FU (siBAP1)	Day 1	Con vs siBAP1#2	5 vs 5	Dunnett's post hoc	2.67 vs 1.47	<0.001	***
Suppl. Fig	Cell viability (CellTiter-Glo)	SNU620/5FU (siBAP1)	Day 2	Overall (one-way ANOVA)	5	One-way ANOVA	F(2,12) = 52.9	<0.001	***
Suppl. Fig	Cell viability (CellTiter-Glo)	SNU620/5FU (siBAP1)	Day 2	Con vs siBAP1#1	5 vs 5	Dunnett's post hoc	4.50 vs 2.52	<0.001	***
Suppl. Fig	Cell viability (CellTiter-Glo)	SNU620/5FU (siBAP1)	Day 2	Con vs siBAP1#2	5 vs 5	Dunnett's post hoc	4.50 vs 2.59	<0.001	***
Suppl. Fig	Cell viability (CellTiter-Glo)	SNU620/5FU (siBAP1)	Day 3	Overall (one-way ANOVA)	5	One-way ANOVA	F(2,12) = 78.2	<0.001	***
Suppl. Fig	Cell viability (CellTiter-Glo)	SNU620/5FU (siBAP1)	Day 3	Con vs siBAP1#1	5 vs 5	Dunnett's post hoc	6.35 vs 3.69	<0.001	***
Suppl. Fig	Cell viability (CellTiter-Glo)	SNU620/5FU (siBAP1)	Day 3	Con vs siBAP1#2	5 vs 5	Dunnett's post hoc	6.35 vs 3.23	<0.001	***
Figure 5D	Cell viability (CellTiter-Glo)	SNU620 (iBAP-II/5FU)	Day 1	Overall (one-way ANOVA)	4–5	One-way ANOVA	F(3,14) = 17.3	<0.001	***
Figure 5D	Cell viability (CellTiter-Glo)	SNU620 (iBAP-II/5FU)	Day 1	5FU vs Con	4 vs 5	Tukey's HSD	2.09 vs 2.32	0.006	**
Figure 5D	Cell viability (CellTiter-Glo)	SNU620 (iBAP-II/5FU)	Day 1	5FU vs iBAP-II	4 vs 5	Tukey's HSD	2.09 vs 1.99	0.295	ns
Figure 5D	Cell viability (CellTiter-Glo)	SNU620 (iBAP-II/5FU)	Day 1	5FU vs iBAP-II+5FU	4 vs 4	Tukey's HSD	2.09 vs 1.97	0.234	ns
Figure 5D	Cell viability (CellTiter-Glo)	SNU620 (iBAP-II/5FU)	Day 1	Con vs iBAP-II	5 vs 5	Tukey's HSD	2.32 vs 1.99	<0.001	***
Figure 5D	Cell viability (CellTiter-Glo)	SNU620 (iBAP-II/5FU)	Day 1	Con vs iBAP-II+5FU	5 vs 4	Tukey's HSD	2.32 vs 1.97	<0.001	***
Figure 5D	Cell viability (CellTiter-Glo)	SNU620 (iBAP-II/5FU)	Day 1	iBAP-II vs iBAP-II+5FU	5 vs 4	Tukey's HSD	1.99 vs 1.97	0.993	ns
Figure 5D	Cell viability (CellTiter-Glo)	SNU620 (iBAP-II/5FU)	Day 2	Overall (one-way ANOVA)	4–5	One-way ANOVA	F(3,14) = 12.8	<0.001	***
Figure 5D	Cell viability (CellTiter-Glo)	SNU620 (iBAP-II/5FU)	Day 2	5FU vs Con	5 vs 4	Tukey's HSD	2.30 vs 3.16	<0.001	***
Figure 5D	Cell viability (CellTiter-Glo)	SNU620 (iBAP-II/5FU)	Day 2	5FU vs iBAP-II	5 vs 5	Tukey's HSD	2.30 vs 2.41	0.864	ns
Figure 5D	Cell viability (CellTiter-Glo)	SNU620 (iBAP-II/5FU)	Day 2	5FU vs iBAP-II+5FU	5 vs 4	Tukey's HSD	2.30 vs 2.40	0.909	ns
Figure 5D	Cell viability (CellTiter-Glo)	SNU620 (iBAP-II/5FU)	Day 2	Con vs iBAP-II	4 vs 5	Tukey's HSD	3.16 vs 2.41	0.001	**
Figure 5D	Cell viability (CellTiter-Glo)	SNU620 (iBAP-II/5FU)	Day 2	Con vs iBAP-II+5FU	4 vs 4	Tukey's HSD	3.16 vs 2.40	0.002	**
Figure 5D	Cell viability (CellTiter-Glo)	SNU620 (iBAP-II/5FU)	Day 2	iBAP-II vs iBAP-II+5FU	5 vs 4	Tukey's HSD	2.41 vs 2.40	1.000	ns
Figure 5D	Cell viability (CellTiter-Glo)	SNU620 (iBAP-II/5FU)	Day 3	Overall (one-way ANOVA)	3–4	One-way ANOVA	F(3,10) = 40.2	<0.001	***
Figure 5D	Cell viability (CellTiter-Glo)	SNU620 (iBAP-II/5FU)	Day 3	5FU vs Con	3 vs 4	Tukey's HSD	3.27 vs 5.58	<0.001	***
Figure 5D	Cell viability (CellTiter-Glo)	SNU620 (iBAP-II/5FU)	Day 3	5FU vs iBAP-II	3 vs 4	Tukey's HSD	3.27 vs 3.89	0.159	ns
Figure 5D	Cell viability (CellTiter-Glo)	SNU620 (iBAP-II/5FU)	Day 3	5FU vs iBAP-II+5FU	3 vs 3	Tukey's HSD	3.27 vs 2.96	0.709	ns
Figure 5D	Cell viability (CellTiter-Glo)	SNU620 (iBAP-II/5FU)	Day 3	Con vs iBAP-II	4 vs 4	Tukey's HSD	5.58 vs 3.89	<0.001	***
Figure 5D	Cell viability (CellTiter-Glo)	SNU620 (iBAP-II/5FU)	Day 3	Con vs iBAP-II+5FU	4 vs 3	Tukey's HSD	5.58 vs 2.96	<0.001	***
Figure 5D	Cell viability (CellTiter-Glo)	SNU620 (iBAP-II/5FU)	Day 3	iBAP-II vs iBAP-II+5FU	4 vs 3	Tukey's HSD	3.89 vs 2.96	0.026	*
Figure 5D	Cell viability (CellTiter-Glo)	SNU620/5FU (iBAP-II/5FU)	Day 1	Overall (one-way ANOVA)	4–5	One-way ANOVA	F(3,15) = 9.3	0.001	**
Figure 5D	Cell viability (CellTiter-Glo)	SNU620/5FU (iBAP-II/5FU)	Day 1	5FU vs Con	5 vs 5	Tukey's HSD	2.02 vs 2.21	0.469	ns
Figure 5D	Cell viability (CellTiter-Glo)	SNU620/5FU (iBAP-II/5FU)	Day 1	5FU vs iBAP-II	5 vs 4	Tukey's HSD	2.02 vs 2.02	1.000	ns
Figure 5D	Cell viability (CellTiter-Glo)	SNU620/5FU (iBAP-II/5FU)	Day 1	5FU vs iBAP-II+5FU	5 vs 5	Tukey's HSD	2.02 vs 1.58	0.013	*
Figure 5D	Cell viability (CellTiter-Glo)	SNU620/5FU (iBAP-II/5FU)	Day 1	Con vs iBAP-II	5 vs 4	Tukey's HSD	2.21 vs 2.02	0.501	ns
Figure 5D	Cell viability (CellTiter-Glo)	SNU620/5FU (iBAP-II/5FU)	Day 1	Con vs iBAP-II+5FU	5 vs 5	Tukey's HSD	2.21 vs 1.58	<0.001	***
Figure 5D	Cell viability (CellTiter-Glo)	SNU620/5FU (iBAP-II/5FU)	Day 1	iBAP-II vs iBAP-II+5FU	4 vs 5	Tukey's HSD	2.02 vs 1.58	0.020	*
Figure 5D	Cell viability (CellTiter-Glo)	SNU620/5FU (iBAP-II/5FU)	Day 2	Overall (one-way ANOVA)	5	One-way ANOVA	F(3,16) = 29.9	<0.001	***
Figure 5D	Cell viability (CellTiter-Glo)	SNU620/5FU (iBAP-II/5FU)	Day 2	5FU vs Con	5 vs 5	Tukey's HSD	2.61 vs 3.23	0.005	**
Figure 5D	Cell viability (CellTiter-Glo)	SNU620/5FU (iBAP-II/5FU)	Day 2	5FU vs iBAP-II	5 vs 5	Tukey's HSD	2.61 vs 2.74	0.852	ns
Figure 5D	Cell viability (CellTiter-Glo)	SNU620/5FU (iBAP-II/5FU)	Day 2	5FU vs iBAP-II+5FU	5 vs 5	Tukey's HSD	2.61 vs 1.79	<0.001	***
Figure 5D	Cell viability (CellTiter-Glo)	SNU620/5FU (iBAP-II/5FU)	Day 2	Con vs iBAP-II	5 vs 5	Tukey's HSD	3.23 vs 2.74	0.027	*
Figure 5D	Cell viability (CellTiter-Glo)	SNU620/5FU (iBAP-II/5FU)	Day 2	Con vs iBAP-II+5FU	5 vs 5	Tukey's HSD	3.23 vs 1.79	<0.001	***
Figure 5D	Cell viability (CellTiter-Glo)	SNU620/5FU (iBAP-II/5FU)	Day 2	iBAP-II vs iBAP-II+5FU	5 vs 5	Tukey's HSD	2.74 vs 1.79	<0.001	***
Figure 5D	Cell viability (CellTiter-Glo)	SNU620/5FU (iBAP-II/5FU)	Day 3	Overall (one-way ANOVA)	3–4	One-way ANOVA	F(3,9) = 42.1	<0.001	***
Figure 5D	Cell viability (CellTiter-Glo)	SNU620/5FU (iBAP-II/5FU)	Day 3	5FU vs Con	3 vs 3	Tukey's HSD	5.68 vs 6.04	0.728	ns
Figure 5D	Cell viability (CellTiter-Glo)	SNU620/5FU (iBAP-II/5FU)	Day 3	5FU vs iBAP-II	3 vs 3	Tukey's HSD	5.68 vs 4.74	0.091	ns
Figure 5D	Cell viability (CellTiter-Glo)	SNU620/5FU (iBAP-II/5FU)	Day 3	5FU vs iBAP-II+5FU	3 vs 4	Tukey's HSD	5.68 vs 2.80	<0.001	***
Figure 5D	Cell viability (CellTiter-Glo)	SNU620/5FU (iBAP-II/5FU)	Day 3	Con vs iBAP-II	3 vs 3	Tukey's HSD	6.04 vs 4.74	0.019	*
Figure 5D	Cell viability (CellTiter-Glo)	SNU620/5FU (iBAP-II/5FU)	Day 3	Con vs iBAP-II+5FU	3 vs 4	Tukey's HSD	6.04 vs 2.80	<0.001	***
Figure 5D	Cell viability (CellTiter-Glo)	SNU620/5FU (iBAP-II/5FU)	Day 3	iBAP-II vs iBAP-II+5FU	3 vs 4	Tukey's HSD	4.74 vs 2.80	0.001	**
Figure 2C	Colony formation	AGS	Endpoint	Overall (one-way ANOVA)	3	One-way ANOVA	F(2,6) = 221.8	<0.001	***
Figure 2C	Colony formation	AGS	Endpoint	Control siRNA vs siBAP1 #1	3 vs 3	Dunnett's post hoc	113.3 vs 44.7	<0.001	***
Figure 2C	Colony formation	AGS	Endpoint	Control siRNA vs siBAP1 #2	3 vs 3	Dunnett's post hoc	113.3 vs 19.3	<0.001	***
Figure 2C	Colony formation	SNU484	Endpoint	Overall (one-way ANOVA)	3	One-way ANOVA	F(2,6) = 126.7	<0.001	***
Figure 2C	Colony formation	SNU484	Endpoint	Control siRNA vs siBAP1 #1	3 vs 3	Dunnett's post hoc	65.3 vs 21.7	<0.001	***
Figure 2C	Colony formation	SNU484	Endpoint	Control siRNA vs siBAP1 #2	3 vs 3	Dunnett's post hoc	65.3 vs 2.3	<0.001	***
Figure 2D	Transwell migration	AGS	Endpoint	Overall (one-way ANOVA)	3	One-way ANOVA	F(2,6) = 73.3	<0.001	***
Figure 2D	Transwell migration	AGS	Endpoint	Control siRNA vs siBAP1 #1	3 vs 3	Dunnett's post hoc	1042.7 vs 383.7	<0.001	***
Figure 2D	Transwell migration	AGS	Endpoint	Control siRNA vs siBAP1 #2	3 vs 3	Dunnett's post hoc	1042.7 vs 416.3	<0.001	***
Figure 2D	Transwell migration	SNU484	Endpoint	Overall (one-way ANOVA)	3	One-way ANOVA	F(2,6) = 182.0	<0.001	***
Figure 2D	Transwell migration	SNU484	Endpoint	Control siRNA vs siBAP1 #1	3 vs 3	Dunnett's post hoc	1005.7 vs 474.0	<0.001	***
Figure 2D	Transwell migration	SNU484	Endpoint	Control siRNA vs siBAP1 #2	3 vs 3	Dunnett's post hoc	1005.7 vs 408.0	<0.001	***
Figure 2E	Transwell invasion	AGS	Endpoint	Overall (one-way ANOVA)	3	One-way ANOVA	F(2,6) = 85.3	<0.001	***
Figure 2E	Transwell invasion	AGS	Endpoint	Control siRNA vs siBAP1 #1	3 vs 3	Dunnett's post hoc	706.7 vs 192.7	<0.001	***
Figure 2E	Transwell invasion	AGS	Endpoint	Control siRNA vs siBAP1 #2	3 vs 3	Dunnett's post hoc	706.7 vs 299.7	<0.001	***
Figure 2E	Transwell invasion	SNU484	Endpoint	Overall (one-way ANOVA)	3	One-way ANOVA	F(2,6) = 91.5	<0.001	***
Figure 2E	Transwell invasion	SNU484	Endpoint	Control siRNA vs siBAP1 #1	3 vs 3	Dunnett's post hoc	404.0 vs 133.0	<0.001	***
Figure 2E	Transwell invasion	SNU484	Endpoint	Control siRNA vs siBAP1 #2	3 vs 3	Dunnett's post hoc	404.0 vs 159.0	<0.001	***
Figure 4C	Apoptosis (Annexin V, %)	AGS	Endpoint	Overall (one-way ANOVA)	3	One-way ANOVA	F(2,6) = 49.0	<0.001	***
Figure 4C	Apoptosis (Annexin V, %)	AGS	Endpoint	Control siRNA vs siBAP1 #1	3 vs 3	Dunnett's post hoc	11.4 vs 55.6	<0.001	***
Figure 4C	Apoptosis (Annexin V, %)	AGS	Endpoint	Control siRNA vs siBAP1 #2	3 vs 3	Dunnett's post hoc	11.4 vs 63.6	<0.001	***
Figure 4C	Apoptosis (Annexin V, %)	SNU484	Endpoint	Overall (one-way ANOVA)	3	One-way ANOVA	F(2,6) = 55.4	<0.001	***
Figure 4C	Apoptosis (Annexin V, %)	SNU484	Endpoint	Control siRNA vs siBAP1 #1	3 vs 3	Dunnett's post hoc	15.1 vs 55.9	<0.001	***
Figure 4C	Apoptosis (Annexin V, %)	SNU484	Endpoint	Control siRNA vs siBAP1 #2	3 vs 3	Dunnett's post hoc	15.1 vs 59.3	<0.001	***
Figure 4D	Apoptosis (Annexin V, %)	AGS	Endpoint	Vehicle vs iBAP-II	3 vs 3	Two-sided Student's t-test	t(4) = 7.05	0.002	**
Figure 4D	Apoptosis (Annexin V, %)	SNU484	Endpoint	Vehicle vs iBAP-II	3 vs 3	Two-sided Student's t-test	t(4) = 9.95	<0.001	***
Figure 5E	Apoptosis (Annexin V, %)	SNU620	Endpoint	Overall (one-way ANOVA)	3	One-way ANOVA	F(3,8) = 70.4	<0.001	***
Figure 5E	Apoptosis (Annexin V, %)	SNU620	Endpoint	5-FU vs Vehicle	3 vs 3	Tukey's HSD	38.3 vs 9.1	<0.001	***
Figure 5E	Apoptosis (Annexin V, %)	SNU620	Endpoint	5-FU vs iBAP-II	3 vs 3	Tukey's HSD	38.3 vs 20.6	0.008	**
Figure 5E	Apoptosis (Annexin V, %)	SNU620	Endpoint	5-FU vs iBAP-II + 5-FU	3 vs 3	Tukey's HSD	38.3 vs 62.1	0.001	**
Figure 5E	Apoptosis (Annexin V, %)	SNU620	Endpoint	Vehicle vs iBAP-II	3 vs 3	Tukey's HSD	9.1 vs 20.6	0.071	ns
Figure 5E	Apoptosis (Annexin V, %)	SNU620	Endpoint	Vehicle vs iBAP-II + 5-FU	3 vs 3	Tukey's HSD	9.1 vs 62.1	<0.001	***
Figure 5E	Apoptosis (Annexin V, %)	SNU620	Endpoint	iBAP-II vs iBAP-II + 5-FU	3 vs 3	Tukey's HSD	20.6 vs 62.1	<0.001	***
Figure 5E	Apoptosis (Annexin V, %)	SNU620/5-FU	Endpoint	Overall (one-way ANOVA)	3	One-way ANOVA	F(3,8) = 795.3	<0.001	***
Figure 5E	Apoptosis (Annexin V, %)	SNU620/5-FU	Endpoint	5-FU vs Vehicle	3 vs 3	Tukey's HSD	13.8 vs 13.0	0.964	ns
Figure 5E	Apoptosis (Annexin V, %)	SNU620/5-FU	Endpoint	5-FU vs iBAP-II	3 vs 3	Tukey's HSD	13.8 vs 28.4	<0.001	***
Figure 5E	Apoptosis (Annexin V, %)	SNU620/5-FU	Endpoint	5-FU vs iBAP-II + 5-FU	3 vs 3	Tukey's HSD	13.8 vs 82.3	<0.001	***
Figure 5E	Apoptosis (Annexin V, %)	SNU620/5-FU	Endpoint	Vehicle vs iBAP-II	3 vs 3	Tukey's HSD	13.0 vs 28.4	<0.001	***
Figure 5E	Apoptosis (Annexin V, %)	SNU620/5-FU	Endpoint	Vehicle vs iBAP-II + 5-FU	3 vs 3	Tukey's HSD	13.0 vs 82.3	<0.001	***
Figure 5E	Apoptosis (Annexin V, %)	SNU620/5-FU	Endpoint	iBAP-II vs iBAP-II + 5-FU	3 vs 3	Tukey's HSD	28.4 vs 82.3	<0.001	***

Supplementary Table S1C

Supplementary Table S1C. Number of independent biological replicates per experiment

Figure	Assay	Cell line / sample	n (independent experiments)	Statistical test
Figure 1A	Paired normal/tumour western blot	Gastric cancer patients	20 paired specimens	Wilcoxon signed-rank test
Figure 2B	Cell viability (CellTiter-Glo)	AGS, SNU484	5	Two-way RM ANOVA + one-way ANOVA/Dunnett
Figure 2C	Colony formation	AGS, SNU484	3	One-way ANOVA + Dunnett
Figure 2D	Transwell migration	AGS, SNU484	3	One-way ANOVA + Dunnett
Figure 2E	Transwell invasion	AGS, SNU484	3	One-way ANOVA + Dunnett
Figure 3A–H	IP / IF / CHX chase / ubiquitination	HEK293T, AGS, SNU484	3	Representative of 3 independent experiments
Figure 4A–B	Western blot (apoptotic markers)	AGS, SNU484	3	Representative of 3 independent experiments
Figure 4C	Apoptosis (Annexin V/PI)	AGS, SNU484	3	One-way ANOVA + Dunnett
Figure 4D	Apoptosis (Annexin V/PI)	AGS, SNU484	3	Two-sided Student's t-test (two groups)
Figure 5A–C	Western blot	SNU620, SNU620/5-FU	3	Representative of 3 independent experiments
Figure 5D	Cell viability (CellTiter-Glo)	SNU620, SNU620/5-FU	3–5	Two-way ANOVA + one-way ANOVA/Tukey
Figure 5E	Apoptosis (Annexin V/PI)	SNU620, SNU620/5-FU	3	One-way ANOVA + Tukey
Suppl. Fig 2A	Cell viability (CellTiter-Glo)	SNU620, SNU620/5-FU	5	Two-way RM ANOVA + one-way ANOVA/Dunnett

Supplementary Table S2A

Supplementary Table S2A. Association of BAP1 and BIRC5 expression with overall survival in gastric cancer

Kaplan–Meier plotter (kmplot.com), pooled gastric cancer microarray cohorts, n = 875.
Overall survival; log-rank test. Figure 1C was generated with the tool's optimised cut-point; the pre-specified median split is shown as a robustness analysis. Probe 201419_at corresponds to BAP1 (BRCA1-associated protein 1, Entrez 8314).

Gene	Probe set	Cohort / cut-point	n (low / high)	HR (95% CI)	Log-rank P	Median OS, low (months)	Median OS, high (months)
**BAP1**	**201419_at**	**All patients, optimised cut-point (as shown in Figure 1C)**	**242 / 633**	**1.69 (1.38–2.08)**	**3.7×10^{-7}**	**65.0**	**24.6**
**BAP1**	**201419_at**	**All patients, pre-specified median split**	**439 / 436**	**1.49 (1.25–1.76)**	**4.8×10^{-6}**	**44.1**	**23.6**
BIRC5	202095_s_at	All patients, pre-specified median split	438 / 437	0.88 (0.74–1.04)	0.144	28.8	28.3

Supplementary Table S2B

Supplementary Table S2B. BAP1 (201419_at) and overall survival, stratified by clinicopathological variable

Kaplan–Meier plotter; pre-specified median split applied within each stratum; overall survival; log-rank test.
Multivariable Cox regression is not supported by this resource, so stratified analyses are presented instead:
each stratum is internally homogeneous for the stratifying variable, so the association within it cannot be explained by that variable.
Estimates in the smallest strata are imprecise.
Corresponds to Supplementary Figure S3.

Subgroup	n	HR (95% CI)	Log-rank P	Median OS, low (months)	Median OS, high (months)
**◗ All patients**	**875**	**1.49 (1.25–1.76)**	**4.8×10^{-6}**	**44.1**	**23.6**
Stage I	69	2.27 (0.77–6.63)	0.125	80.7	45.1
Stage II	145	0.92 (0.50–1.69)	0.794	45.8	40.2
Stage III	319	1.56 (1.17–2.07)	0.0024	37.5	24.5
Stage IV	152	1.12 (0.77–1.65)	0.553	16.8	16.3
N0	76	1.51 (0.61–3.73)	0.370	80.7	45.1
N1–3	437	1.44 (1.11–1.88)	0.006	76.2	29.2
M0	459	1.47 (1.11–1.95)	0.0064	25.2	17.4
T3	208	1.45 (1.03–2.06)	0.033	28.1	19.2
Female	244	1.44 (1.01–2.04)	0.042	65.0	25.5
Male	566	1.71 (1.37–2.12)	9.2×10^{-7}	39.8	21.0
Lauren, intestinal	336	2.06 (1.49–2.85)	8.6×10^{-6}	23.4	14.2
Lauren, diffuse	248	1.08 (0.77–1.51)	0.671	36.4	35.9

Supplementary Table S2C

Supplementary Table S2C. BAP1 and BIRC5 and overall survival, stratified by adjuvant treatment

Kaplan–Meier plotter; pre-specified median split applied within each treatment subgroup; overall survival; log-rank test.
The test for interaction compares the log hazard ratios of the 5-FU–based adjuvant and surgery-alone subgroups (Wald test on the difference).
Treatment was not randomised and patients receiving adjuvant chemotherapy had more advanced disease, so confounding by indication cannot be excluded; these analyses are hypothesis-generating. Corresponds to Supplementary Figure S4.

Gene	Treatment subgroup	n	HR (95% CI)	Log-rank P	Median OS, low (months)	Median OS, high (months)
BAP1	◗ All patients	875	1.49 (1.25–1.76)	4.8×10^{-6}	44.1	23.6
BAP1	5-FU–based adjuvant	157	1.11 (0.79–1.57)	0.542	13.0	11.3
BAP1	Surgery alone	393	1.03 (0.77–1.37)	0.867	53.4	52.6
BAP1	Other adjuvant	80	3.70 (1.34–10.20)	0.007	not reached	—
BIRC5	◗ All patients	875	0.88 (0.74–1.04)	0.144	28.8	28.3
BIRC5	5-FU–based adjuvant	157	1.73 (1.22–2.45)	0.0019	15.2	8.1
BIRC5	Surgery alone	393	0.81 (0.60–1.07)	0.141	44.6	76.2
BIRC5	Other adjuvant	80	0.82 (0.34–1.98)	0.654	31.3	77.2

**Test for interaction, 5-FU–based adjuvant versus surgery alone**			**Ratio of HRs (95% CI)**	**P**		
BAP1			1.08 (0.69–1.69)	0.744		
BIRC5			2.14 (1.36–3.36)	0.001		
